# PA1 participates in the maintenance of blood–testis barrier integrity via cooperation with JUN in the Sertoli cells of mice

**DOI:** 10.1186/s13578-022-00773-y

**Published:** 2022-04-04

**Authors:** Bo Liu, Chao Liu, Binfang Ma, Ruidan Zhang, Zhiwei Zhao, Sai Xiao, Wanjun Cao, Yanjie Ma, Guozhang Zhu, Wei Li, Zhen Li

**Affiliations:** 1 Department of Human Anatomy, Histology and Embryology, The Fourth Military Medical University, Xi’an, 710032 China; 2 Institute of Reproductive Health and Perinatology, Guangzhou Women and Children’s Medical Center, Guangzhou, 510000 China; 3 University of the Chinese Academy of Sciences, Beijing, 100049 China; 4 Department of Biology, Marshall University, Huntington, WV 25755 USA

**Keywords:** PA1, Sertoli cell, Blood–testis-barrier, JUN, Connexin43

## Abstract

**Background:**

The blood–testis barrier (BTB) is essential to the microenvironment of spermatogenesis, and Sertoli cells provide the cellular basis for BTB construction. Numerous nuclear transcription factors have been identified to be vital for the proper functioning of Sertoli cells. PA1 has been reported to play important roles during diverse biological processes, yet its potential function in male reproduction is still unknown.

**Results:**

Here, we show that PA1 was highly expressed in human and mouse testis and predominantly localized in the nuclei of Sertoli cells. Sertoli cell-specific *Pa1* knockout resulted in an azoospermia-like phenotype in mice. The knockout of this gene led to multiple defects in spermatogenesis, such as the disorganization of the cytoskeleton during basal and apical ectoplasmic specialization and the disruption of the BTB. Further transcriptomic analysis, together with Cut-Tag results of PA1 in Sertoli cells, revealed that PA1 could affect the expression of a subset of genes that are essential for the normal function of Sertoli cells, including those genes associated with actin organization and cellular junctions such as *Connexin43* (*Cx43*). We further demonstrated that the expression of Cx43 depended on the interaction between JUN, one of the AP-1 complex transcription factors, and PA1.

**Conclusion:**

Overall, our findings reveal that PA1 is essential for the maintenance of BTB integrity in Sertoli cells and regulates BTB construction-related gene expression via transcription factors. Thus, this newly discovered mechanism in Sertoli cells provides a potential diagnostic or even therapeutic target for some individuals with azoospermia.

**Supplementary Information:**

The online version contains supplementary material available at 10.1186/s13578-022-00773-y.

## Introduction

The differentiation and maintenance of some tissues require special niches that need to exclude macromolecules from these tissues, and all these processes are dependent on the blood–tissue barrier. General examples for barriers are the blood–brain-, the blood–retina-, the blood–placenta- and the blood–testis barrier (BTB) [1–4]. Among these barriers, the BTB exhibits unique structural and functional characteristics, significantly different from the other blood–tissue barriers. Rather than performing in a relatively stable state, typical of most tissue barriers, the BTB provides a distinct, dynamic, and elaborate barrier established during seminiferous epithelium cycle [4]. BTB formation solely depends on adjacent Sertoli cells, which are responsible for compartmentalizing the seminiferous epithelium. Other blood–tissue barriers rely on vascular endothelial cells in conjunction with other types of cells. For example, vascular endothelial cells together with adjacent surrounding cells of the neurovascular unit contribute to the blood–brain barrier (BBB) [5]. Numerous studies have proven the BTB is indispensable for normal spermatogenesis [6–9].

Tight junctions (TJs), adherent junctions (AJs), gap junctions and other junctional proteins, together constitute the highly specialized BTB, which is precisely regulated by intricate signals within Sertoli cells [4]. The molecular basis of the BTB is mainly comprised of three types of proteins: integral membrane proteins, adaptor proteins and cytoskeletal proteins [5]. As for TJs, transmembrane proteins, such as Occludin or Claudin11, form a homophilic protein–protein interlocking structure at the intercellular space and are connected to the actin cytoskeleton via the interaction between adaptor proteins, including ZO-1/2/3 and the cytoplasmic domains of these transmembrane proteins [10, 11]. Like the TJs, cadherins bind to α- and β‐Catenin, anchoring the cytoskeleton to form AJs [12]. As for gap junctions, connexins expressed on cell membranes connect to each other, working as communication channels for adjacent cells [13]. It has been demonstrated that even the absence of a single constituent protein localized in the BTB could lead to aberrant spermatogenesis and male infertility. The global knockout of *Occludin* or *Claudin11* in mice has been shown to lead to the disruption of seminiferous tubules and the progressive germ cell loss [8, 9]. The specific knockout of *Cx43* in Sertoli cells resulted in disorganized intercellular junctional complexes between adjacent cells, ultimately leading to the failure of spermatogenesis [7, 14]. Therefore, precisely controlled BTB formation is required for normal spermatogenesis.

In addition to Sertoli cells’ essential role in the construction of the BTB, these somatic cells in the seminiferous epithelium exert their pivotal functions by forming multiple types of cell junctions with germ cells [4, 15]. At the apical surface of Sertoli cells, highly specialized cell–cell junctions called apical ectoplasmic specialization (aES) between the Sertoli cells and elongated spermatids, is comprised of F-actin bundles, adaptor proteins and other cell junctional components [6]. The normal organization and arrangement of aES have been proved to be indispensable for spermatid development and the destruction of the structure leads to some defects of spermiogenesis [6, 16]. Overall, the proper functions of Sertoli cells are indispensable for the success of spermatogenesis.

Plenty of nuclear transcription factors or co-factors that have been identified in Sertoli cells are crucial for the physiological functions of Sertoli cells. For example, in adult testis, depletion of Wilms Tumor (WT1), a specific transcription factor of Sertoli cells, will disrupt the BTB and the polarity of Sertoli cells, leading to infertility [17]. In addition, sex-determining region Y-box8 (*Sox8*)-knockout mice presented remarkable germ cell loss and defective BTB [18], and deletion of Androgen Receptor (AR) in Sertoli cells caused meiosis arrest of spermatocytes at the diplotene stage [7]. Moreover, the specific knockout of Adenomatous polyposis coli protein (*Apc*) in Sertoli cells was shown to destroy the BTB, resulting in a loss of premature germ cells [19]. Although these findings provide a glimpse into the role of nuclear transcription factors or cofactors involved in the regulation of Sertoli cell functions, many other factors remain to be identified and understood.

Pax transactivation domain-interacting protein (PTIP) associated protein 1 (PA1) was first characterized as a protein interacting with PTIP within mixed-lineage leukemia 3/4 (MLL3/4) complexes [20]. Later reports showed that PA1 engaged in in many biological processes during development and differentiation. First, PA1 participates in early embryo development. Loss of PA1 in embryos leads to severe developmental defects that occur in E8.0 and no homozygous mutant has survived to E10.5, most likely due to decreased expression of BMP2 [21]. Recently, PA1 was found to be expressed in the human fetal cerebral cortex, and a homozygous missense mutation (c.274A > G; p.Ser92Gly, NM_024516.4) was found to be associated with severe neurodevelopmental disorders [22, 23]. In addition, PA1 has been found to cooperate with phosphorylated CREB (pCREB) and glucocorticoid receptor (GR) to regulate the expression of key adipose differentiation transcription factors C/EBPβ and C/EBPδ, and the deletion of *Pa1* in white and brown preadipocytes has been shown to severely disrupt adipogenesis [24]. Previous studies have demonstrated that PTIP, which is closely correlated with PA1, is indispensable for male fertility [25]. Together, these findings suggest that PA1 may play critical roles in male reproductive processes.

In our study, we first determined PA1 was mainly expressed in the Sertoli cells of human and mouse testis and elucidated PA1’s indispensable role during spermatogenesis in mouse testis. The deficiency of PA1 in Sertoli cells led to notable germ cell loss, disorganized cytoskeleton both at the apical and basal compartments of seminiferous tubules, and disrupted the BTB. Further analysis revealed that PA1 was necessary for the transcription of a subset of genes that are vital for proper Sertoli cell functioning, including those genes associated with cell-adhesion and cytoskeleton organization, such as *Cx43*. In addition, we find that PA1 cooperates with JUN to regulate *Cx43*, whose disruption also results in male infertility, resembling that of *Amh-pa1*^*−/−*^ mice. Thus, our work uncovers a novel function for PA1 in Sertoli cells, regulating the integrity of the BTB by interacting with JUN to promote the transcription of a specific profile of BTB construction-related genes.

## Results

### PA1 protein is predominantly expressed in Sertoli cells of mouse and human

**Fig. 1:**
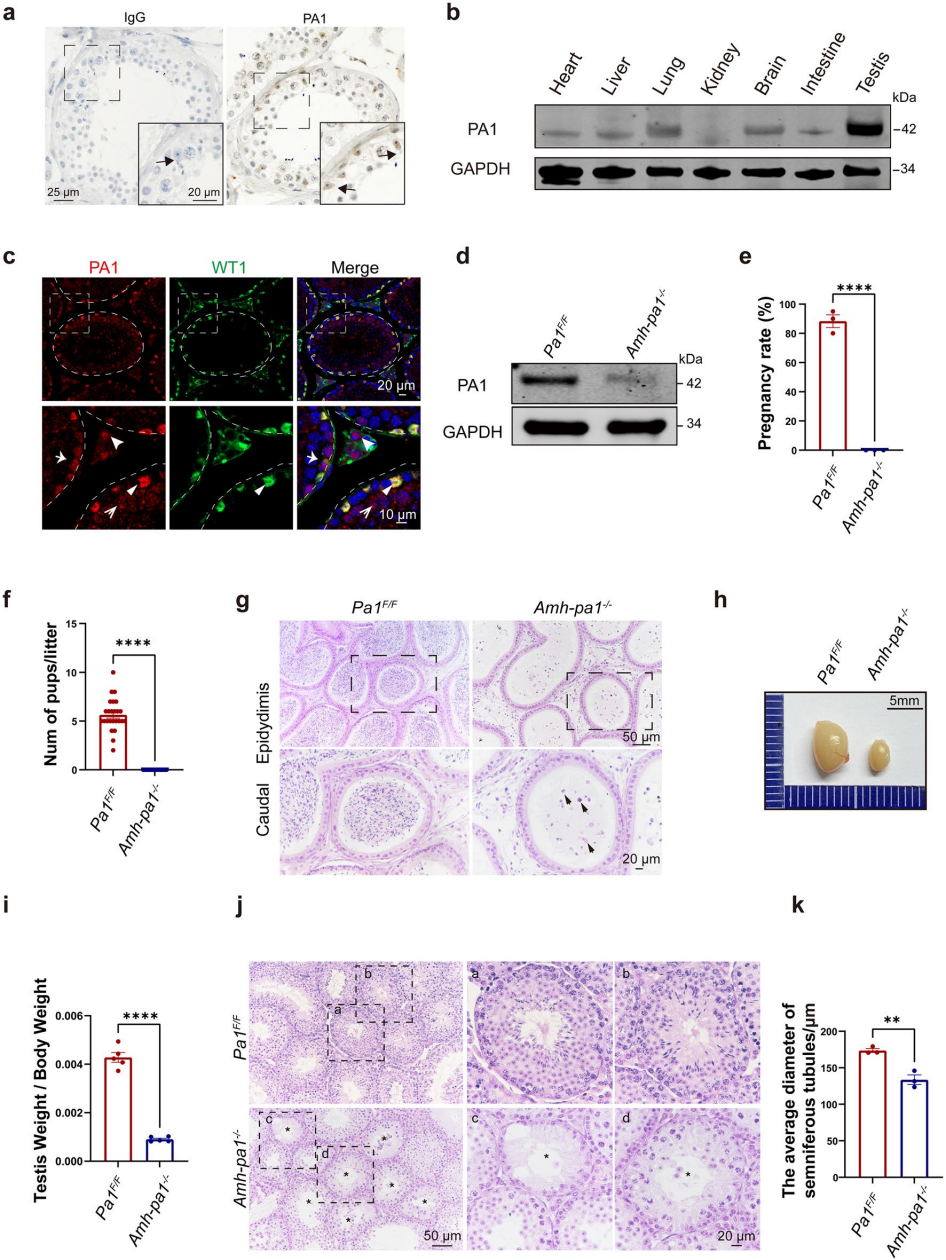
Specific knockout of *Pa1* in Sertoli cells impairs spermatogenesis. **a** PA1 was predominantly localized in the nucleus of human Sertoli cells. Immunohistochemistry (IHC) image of PA1 in normal adult human testis. Dashed boxes showed localization of the enlarged images. Arrows indicated the nuclei of Sertoli cells. **b** PA1 was highly expressed in testis compared with other murine organs. The expression profile of PA1 in different murine organs (8-week-old) using immunoblot. **c** PA1 mainly localized in the nuclei of Sertoli cells in mouse testis. Seminiferous tubule sections of wild-type testes (5-week-old) were co-stained for Sertoli cell marker WT1 (green), PA1 (red) and DAPI (blue). Enlarged images are shown on the lower panel. Dashed circle and curve indicated basal membrane of seminiferous tubules. Arrows represents cells that are positive for PA1 and/or WT1. , Leydig cell; , SPG; , SPC; , SC. **d** PA1 protein levels dramatically decreased in the testes (8-week-old) of *Amh-pa1*^*−/−*^ mice. Immunoblotting of PA1 in *Pa1*^*F/F*^ and *Amh-pa1*^*−/*−^ testes. GAPDH as the loading control. **e** 8-week-old *Amh-pa1*^*−/−*^ mice could not fertilize the female mice. After mating, 88.33 ± 4.41% of wild-type female mice were pregnant after crossing with *Pa1*^*F/F*^ males, while none of wild-type female mice crossing with *Amh-pa1*^*−/−*^ mice became pregnant. (n = 3). Data are presented as mean ± SEM. ****p < 0.0001. **f** The average litter size of *Pa1*^*F/F*^ males and *Amh-pa1*^*−/−*^ mice at 8-week-old (n = 3 independent experiments). Data are presented as mean ± SEM. ****p < 0.0001. **g** Histological analysis of the caudal epididymis of *Pa1*^*F/F*^ and *Amh-pa1*^*−/−*^ mice. Enlarged images are shown in the lower panel. Dashed boxes showed localization of the enlarged images. Debris of immature spermatozoa are indicated with black arrows. **h** The volume of testes of 8-week-old *Amh-pa1*^*−/−*^ mice was significantly smaller than that of *Pa1*^*F/F*^ mice. **i** The ratio of testis weight/body weight of *Amh-pa1*^*−/−*^ mice was significantly lower than that of *Pa1*^*F/F*^ mice (n = 5). Data are presented as mean ± SEM. ****p < 0.0001. **j** Histological analysis of the testis of 8-week-old *Pa1*^*F/F*^ and *Amh-pa1*^*−/−*^ mice. (*a, b*) Two seminiferous tubules are shown for the *Pa1*^*F/F*^ mice with enlarged images shown on the right. (*c*) An empty seminiferous tubule is shown for the *Amh-pa1*^*−/−*^ mice with an enlarged image shown on the right. The enlarged image shows an empty tubule indicated with an asterisk. Germ cells could not be found in these seminiferous tubules. (*d*) A degenerated seminiferous tubule is shown for the *Amh-pa1*^*−/−*^ mice with an enlarged image shown on the right. The enlarged image shows a degenerated tubule indicated with an asterisk. Decreased germ cells are found in these seminiferous tubules. **k** The average diameter of seminiferous tubules of 8-week-old *Amh-pa1*^*−/−*^ mice were significantly lower than those of 8-week-old *Pa1*^*F/F*^ mice. (n = 3). Data are presented as mean ± SEM. **p < 0.01

According to the Human Proteomics Database, PA1 exhibits high expression in human testis, implying a potential role during normal spermatogenesis [26–28]. Therefore, we first investigated the expression and localization of PA1 in normal human testis. Immunohistochemistry results showed that PA1 was predominantly expressed in the nuclei of Sertoli cells (Fig. 1a). To further explore PA1’s potential function, we examined its expression pattern in different tissues of 8-week-old adult mice and found that PA1 was ubiquitously expressed in different tissues yet predominantly expressed in the testis (Fig. 1b). Using mouse testis lysates prepared from mice of varying age, immunoblotting results showed that PA1 could be detected in testis as early as postnatal day 7 (D7), gradually increased until it leveled off at D21, and slightly decreased after D28 (Additional file 3: Fig. S1a). Consistent with the immunoblot results, semi-quantitative PCR results confirmed that PA1 was enriched in the testis, with increases coinciding with the testis development (Additional file 3: Fig. S1b and c).

Immunohistochemistry (IHC) of PA1 in adult mice testis showed that PA1 was predominantly localized in Sertoli cells in all stages of the spermatogenic cycle (Additional file 1: Fig. S1d). However, the expression level of PA1 in Sertoli cells varied in different stages. PA1 was highly expressed in the nuclei of Sertoli cells around the VII-VIII stage and presented relative low expression in Sertoli cells in other stages. To get a more complete picture, we collected different types of germ cells including spermatogonium (SPG), spermatocyte (SPC), round spermatid (rST) and elongated spermatid (eST) from adult mice testes, as well as Sertoli cells. Immunoblotting of PA1 in these cells showed that PA1 was abundant in Sertoli cells while variable expression levels were prevalent in germ cells at different stages (Additional file 1: Fig. S1e). Immunofluorescence also showed that PA1 colocalized with WT1, a Sertoli cell nuclear marker, in wild-type adult mice testes. Weaker PA1 signals could also be seen in the nuclei of Leydig cells and germ cells (Fig. 1c), suggesting that the protein might have potential functions in different testicular cells.

### Sertoli cell-specific knockout of *Pa1* results in azoospermia

To uncover the function of PA1 in Sertoli cells, we obtained *floxed Pa1* mice from the laboratory of Dr. Kai Ge [24]. The *Pa1 floxed* mice were then crossed with *Amh-cre* mice which express Cre recombinase only in the Sertoli cells (Additional file 4: Fig. S2a). These Sertoli cell-specific *Pa1* knockout mice (*Amh-pa1*^*−/−*^) were further confirmed with PCR results. The presence of a 250 bp band indicated floxed *Pa1,* the 200 bp band indicated *Pa1*, and the 400 bp band indicated *Amh-cre* (Additional file 4: Fig. S2b). Immunoblotting was used to confirm the knockout efficiency of *Pa1* in the *Amh-pa1*^*−/−*^ testis. The protein level of PA1 was significantly decreased in *Amh-pa1*^*−/−*^ mice testis (Fig. 1d). Immunofluorescence experiments were used to further examine the specific knockout of PA1, and showed an absence of PA1 signal in Sertoli cells and the presence of PA1 signal in Leydig cells and germ cells (Additional file 4: Fig. S2c). Therefore, PA1 is specifically knocked out in the Sertoli cells of *Amh-pa1*^*−/−*^ mice.

Next, we examined the fertility of *Amh-pa1*^*−/−*^ male mice. *Amh-pa1*^*−/−*^ male mice failed to produce any offspring after mating with wild-type adult female mice (Fig. 1e and f), suggesting *Amh-pa1*^*−/−*^ male mice were completely infertile. To determine whether *Amh-pa1*^*−/−*^ male mice could produce any mature sperm in the epididymis, the cauda epididymis was analyzed via hematoxylin and eosin (H&E) staining. Mature spermatozoa could be hardly found while cell debris and immature germ cell were observed in the caudal epididymis of *Amh-pa1*^*−/−*^ mice, suggesting abnormal exfoliation of germ cells from the seminiferous tubules of *Amh-pa1*^*−/−*^ testis.

### Multiple spermatogenic defects in the ***Amh-pa1***^***−/−***^ testis

To provide a possible explanation for the azoospermia phenotype in *Amh-pa1*^*−/−*^ testis, we next examined the weight and volume of *Amh-pa1*^*−/−*^ and *Pa1*^*F/F*^ mice testis. We found that the testis volume, the testis weight, and the ratio of testis weight/body weight of adult *Amh-pa1*^*−/−*^ mice were significantly decreased compared with those of control mice, while the body weight did not show any difference (Fig. 1h and i) (Additional file 4: Fig. S2d and e).

**Fig. 2:**
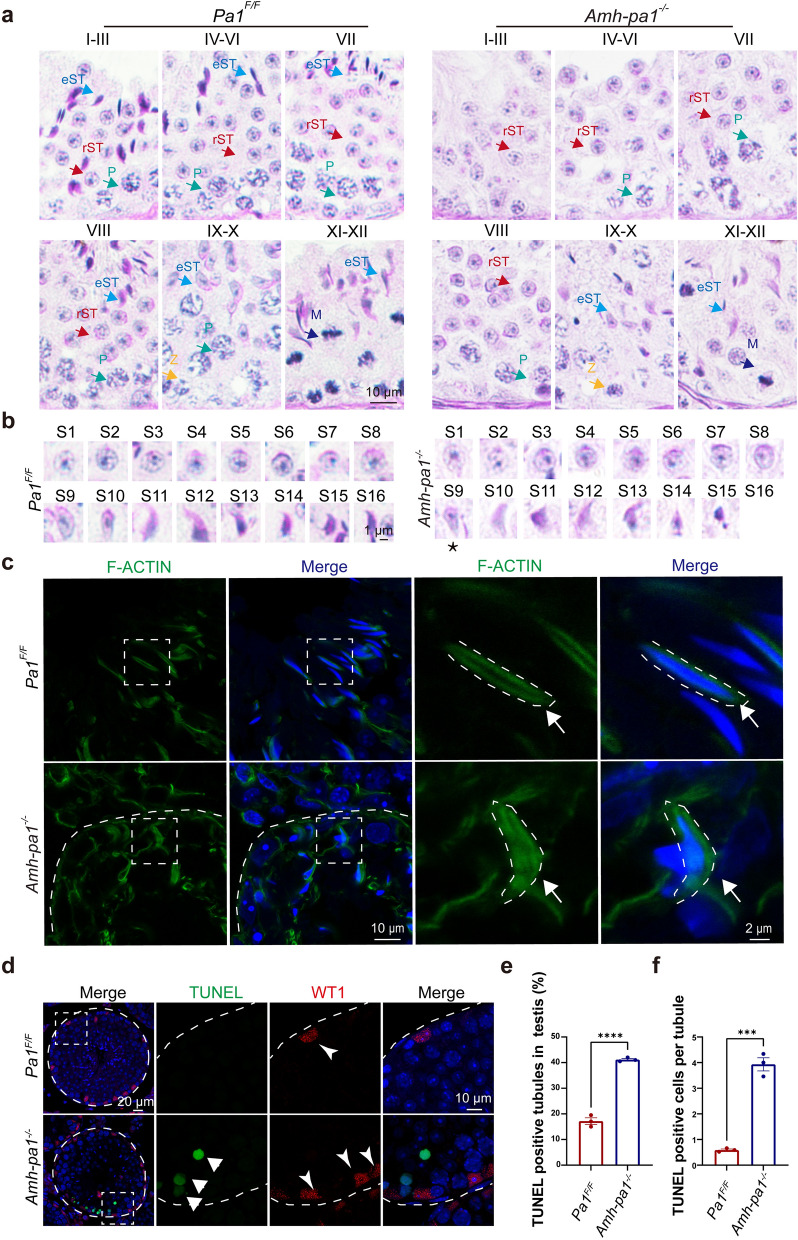
Aberrant spermiogenesis and apical actin cytoskeleton disorganization could be detected in *Amh-pa1*^*−/−*^ mice. **a** PAS staining of testes sections from 8-week-old *Pa1*^*F/F*^ and *Amh-pa1*^*−/−*^ mice. Elongated spermatids were gradually lost along with spermiogenesis. Different colored arrows indicated different types of cells. Z: Zygotene; eST: elongating spermatid; M: meiotic spermatocyte; P: pachytene spermatocyte; rST: round spermatid. **b** PAS staining of spermatids at different developmental stages from 8-week-old *Pa1*^*F/F*^ and *Amh-pa1*^*−/−*^ mice. Deformed spermatid head shaping begins to appear at Step 9 (asterisk) in *Amh-pa1*^*−/−*^ testes. **c** F-actin in the apical compartment of seminiferous tubules was seen to be perturbed and disorganized in adult *Amh-pa1*^*−/−*^ testes. Arrows indicate F-actin bundles. Immunofluorescence using FITC-labeled phalloidin (green) was performed on seminiferous tubules of *Pa1*^*F/F*^ mice (upper) and *Amh-pa1*^*−/−*^ mice (lower). Nuclei were stained with DAPI (blue). Dashed boxes showed localization of the enlarged images. Dashed curve indicated basal membrane of seminiferous tubules in the left images and F-actin structure surrounding the sperm head in the right enlarged images. **d** Seminiferous tubule sections of *Pa1*^*F/F*^ and *Amh-pa1*^*−/−*^ testes were stained for WT1 and TUNEL probe and the nuclei were stained with DAPI. , WT1 positive Sertoli cells (red); , TUNEL positive cells (green). Dashed boxes showed localization of the enlarged images. Dashed circle and curve indicated basal membrane of seminiferous tubules. **e** More TUNEL positive tubules were observed in *Amh-pa1*^*−/−*^ testis compared with *Pa1*^*F/F*^ testis and **f** more TUNEL positive cells per tubule were observed in *Amh-pa1−/−* testis compared with *Pa1F/F* testis. (n = 3). Data are presented as mean ± SEM. ***p < 0.001, ****p < 0.0001

To explore how PA1 in Sertoli cells affect spermatogenesis, we used Periodic acid–Schiff (PAS) staining to examine the stages of spermatogenesis in 8-week-old *Amh-pa1*^*−/−*^ and *Pa1*^*F/F*^ mouse testes. We found that elongating spermatids had abnormal head shaping during spermiogenesis (Fig. 2a). To characterize the morphological defects of *Amh-pa1*^*−/−*^ elongating spermatids, we compared the process of sperm head shaping between *Amh-pa1*^*−/−*^ and *Pa1*^*F/F*^ mice. Remarkably, acrosome and nucleus morphology of step 1 to step 8 proceeded normally in both *Amh-pa1*^*−/−*^ and *Pa1*^*F/F*^ spermatids. The appearance of aberrant spermatid head shaping developed since step 9 in *Amh-pa1*^*−/−*^ mice (Fig. 2b). This aberration became more pronounced at step 11 through 16, suggesting *Pa1* knockout in Sertoli cells lead to defective sperm head development.

Actin filaments, microtubules, and intermediate filaments together constitute the intricate cytoskeleton structure of Sertoli cells. Previous research has reported that the organized actin cytoskeleton of Sertoli cells was indispensable for sperm head shaping [6, 16, 29]. Concentrated within specialized intercellular junctions including ES and tubulobulbar complexes, the actin cytoskeleton is involved in the maintenance of cell–cell junctions and normal spermiogenesis [30].

To determine whether the actin cytoskeleton was aberrant in *Amh-pa1*^*−/−*^ testis, we performed immunofluorescence analysis on testis sections using phalloidin. Images showed that the F-actin structure, which circles the nucleus of elongating spermatids, was disorganized in *Amh-pa1*^*−/−*^ testes but not in wild-type testes (Fig. 2c). In addition, we also detected the disordered arrangement of F-actin bundles at basal compartments of seminiferous tubules (Additional file 5: Fig. S3), implying a disrupted BTB in *Amh-pa1*^*−/−*^ testes. Together, these results indicate the prominent disorganized F-actin structure of *Amh-pa1*^*−/−*^ in Sertoli cells may account for spermiogenesis failure.

### Massive germ cell loss in testes from adult ***Amh-pa1***^***−/−***^ mice

Hematoxylin and eosin staining of testes from adult *Amh-pa1*^*−/−*^ mice revealed empty and degenerated seminiferous tubules (Fig. 1j) that contained only Sertoli cells without any germ cells compared with the control mice. The diameter of the seminiferous tubules in the *Amh-pa1*^*−/−*^ testis was significantly decreased in comparison with those of the control testis (Fig. 1k). Hence, spermatogenesis was disrupted in the *Amh-pa1*^*−/−*^ testis.

Due to the existence of empty tubules in the adult *Amh-pa1*^*−/−*^ testis, we performed immunofluorescence analysis to confirm whether the residual cells were Sertoli cells. Results showed that these tubules were indeed occupied with Sertoli cells (Additional file 6: Fig. S4a). Next, TUNEL assay was performed to detect whether Sertoli cells in the seminiferous tubules were undergoing aberrant apoptosis. Increased positive TUNEL signals were only observed in the *Amh-pa1*^*−/−*^ testis which did not localize in the Sertoli cells (Fig. 2d), indicating that PA1 is dispensable for the survival of Sertoli cells. Both TUNEL positive cells per tubule as well as TUNEL positive tubules were increased in testes from *Amh-pa1*^*−/−*^ mice compared with control mice (Fig. 2e and f), suggesting the loss of germ cells may partially be due to increased germ cell death.

Furthermore, to demonstrate which types of germ cells were affected in the *Amh-pa1*^*−/−*^ seminiferous tubules, several germ cell markers were used to characterize the cells. We first evaluated PCNA, which is a marker of proliferative spermatogonia and primary spermatocytes that can be detected until early pachytene stage, excluding preleptotene spermatocytes [31]. We found PCNA positive cell numbers were reduced in *Amh-pa1*^*−/−*^ testis (Additional file 6: Fig. S4b and e), indicating a loss of spermatocytes and spermatogonia. DDX4-positive germ cells, including spermatocytes and round spermatids, were also reduced in *Amh-pa1*^*−/−*^ testis (Additional file 6: Fig. S4c and f). Moreover, the immunofluorescence results of PLZF, a marker of undifferentiated spermatogonia, showed the exhaustion of these PLZF-positive cells in *Amh-pa1*^*−/−*^ testis (Additional file 6: Fig. S4d and g), suggesting the disruption of normal spermatogonia self-renewal or differentiation. Besides, the immunofluorescence of spermatocyte nuclear spreads did not reveal remarkable abnormalities in meiosis I prophase using γH2AX and SYCP3 antibodies (Additional file 6: Fig. S4h). So, the loss of PA1 in Sertoli cells compromised the survival of different types of germ cells.

### Disrupted BTB integrity in ***Amh-pa1***^***−/−***^ mice

**Fig. 3:**
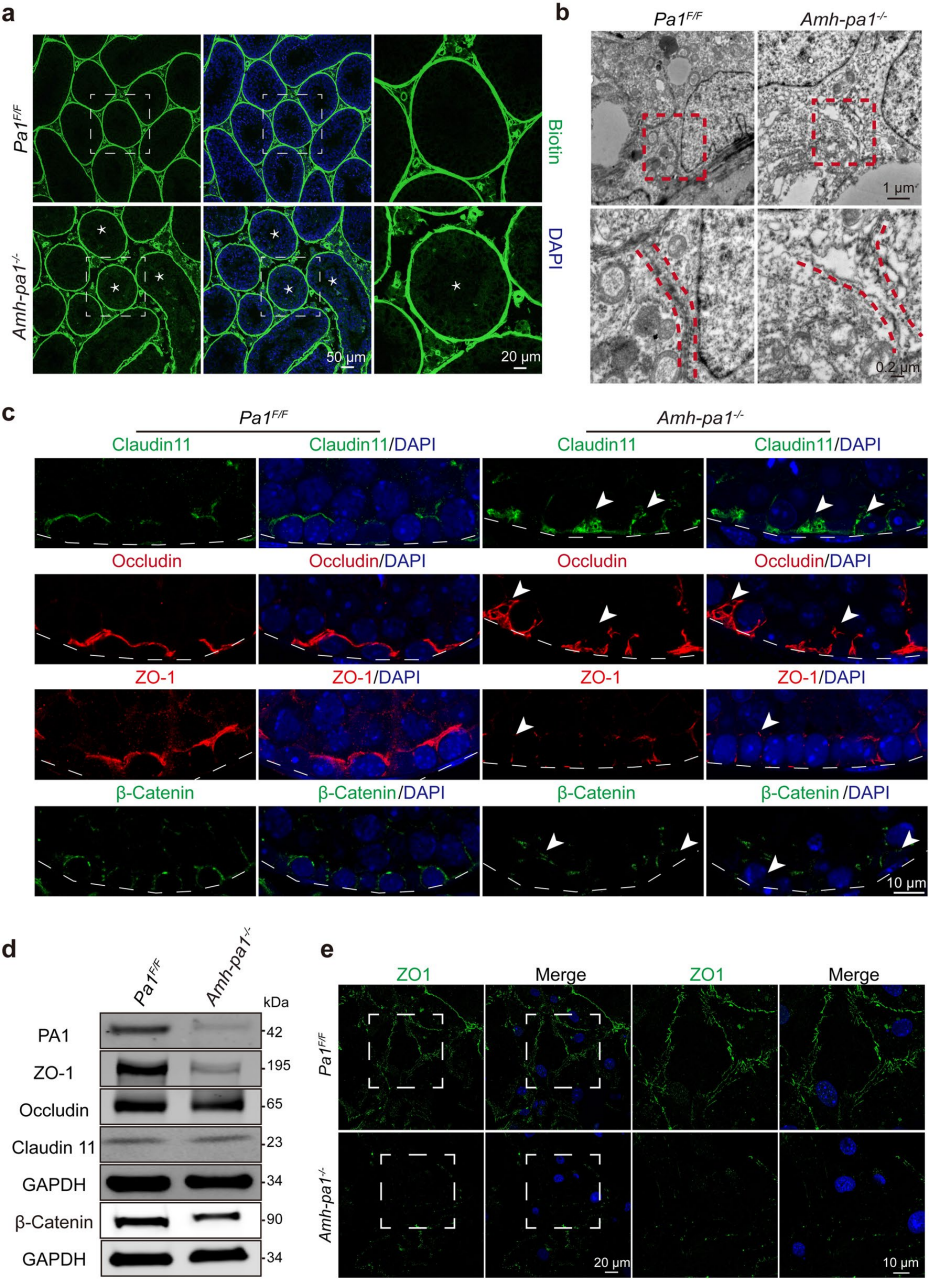
The integrity of BTB is disrupted in *Amh-pa1*^*−/−*^ mice. **a** The integrity of the BTB was observed to be destroyed in 8-week-old *Amh-pa1*^*−/−*^ testes. Immunofluorescence using FITC-labeled streptavidin (green) was conducted on seminiferous tubules of *Pa1*^*F/F*^ (upper) and *Amh-pa1*^*−/−*^ testes (lower) which had been injected with biotin beneath the albuginea. Nuclei were stained with DAPI (blue). Dashed boxes showed localization of the enlarged images. Asterisks indicated biotin penetrated seminiferous tubules. **b** Transmission electron microscope images of adult *Pa1*^*F/F*^ and *Amh-pa1*^*−/−*^ mice testes showing the cleft junctions between Sertoli cells at the basal compartment of seminiferous tubules. Red dashed boxes showed localization of the enlarged images shown in the bottom row. Red dashed lines in the enlarged images indicate the cell–cell junctions. **c** BTB components are shown to be disorganized in 8-week-old *Amh-pa1*^*−/−*^ mice testis. Immunofluorescence analysis using anti-Claudin11, anti-Occludin, anti-ZO-1, and anti-β-catenin was conducted in the testis of *Pa1*^*F/F*^ mice (left) and *Amh-pa1*^*−/−*^ mice (right). Nuclei were stained with DAPI (blue). Arrowheads indicate the aberrant patches or diminished signals of the BTB components. Dashed curve indicated basal membrane of seminiferous tubules. **d** Immunoblot analysis of BTB components, including ZO-1, Occludin, Claudin11, and β-Catenin, in the testes of adult wild-type mice and *Amh-pa1*^*−/−*^ mice. **e** Cell junction cannot be formed between *Amh-pa1*^*−/−*^ Sertoli cells. Immunofluorescence using anti-ZO-1 (green) was conducted on Sertoli cells of *Pa1*^*F/F*^ (upper) and *Amh-pa1*^*−/−*^ testes (lower). Nuclei were stained with DAPI (blue). Dashed boxes showed localization of the enlarged images

Previous studies have demonstrated that a disrupted BTB will lead to a remarkable loss in germ cells and complete infertility of male mice [8, 9]. To further determine whether PA1 is required to maintain the BTB, we assessed the integrity of the BTB in *Amh-pa1*^*−/−*^ testes. After injecting biotin into the testis interstitial tissue, we found that biotin penetrated the lumen of seminiferous tubules of *Amh-pa1*^*−/−*^* mice* while biotin was restricted to the basal part of the seminiferous tubules of control mice (Fig. 3a), suggesting the disruption of the barrier function of the BTB. Next, we performed electron microscopy to visualize the cell junctions formed between Sertoli cells. Discontinuous clefts or gaps could be seen at basal Sertoli-Sertoli cell junctions in *Amh-pa1*^*−/−*^ testis while several electron dense areas could be seen at junctions in control testis, indicating the intact tight junctions (Fig. 3b).

To clarify whether BTB components were also disrupted in *Amh-pa1*^*−/−*^ testis, we performed immunofluorescent staining of Claudin-11, ZO-1, Occludin, and β-Catenin. In control testicular sections, Claudin-11, ZO-1, Occludin, and β-Catenin were distributed orderly at the basal compartment of seminiferous tubules (Fig. 3c), consistent with results from previous studies. However, in *Amh-pa1*^*−/−*^ testicular sections, an abnormal localization pattern was detected. With Claudin11 and Occludin, we found large patches or clumps of the proteins concentrated at basal compartments (Fig. 3c). With ZO-1 and β-Catenin, weaker signals were found at basal compartments in some of the tubules while some tubules still presented aggregated and patchy signals (Fig. 3c). Further immunoblot analyses of *Amh-pa1*^*−/−*^ testes showed that Claudin 11 and Occludin expression was similar to that in control testes, whereas ZO-1 and β-Catenin were reduced significantly. These results are consistent with previous immunofluorescence results in *Amh-pa1*^*−/−*^ testes, indicating a loss of PA1 in Sertoli cells may influence the BTB components (Fig. 3d).

To further confirm whether PA1 deficiency would affect the formation of cell junctions in Sertoli cells, immunofluorescent staining of ZO1 was performed on purified Sertoli cell obtained from the testes of 20-day-old mice. In control Sertoli cells, ZO1 localized mainly at the junctions between adjacent cells, whereas in *Amh-pa1*^*−/−*^ Sertoli cells, adjacent cells could not form boundary junctions (Fig. 3e), implying PA1 deficiency in Sertoli cells compromises their ability to form functional cell junctions. Hence, *Pa1* knockout in Sertoli cells could disturb the integrity of the BTB and disrupt the distribution of BTB components.

### PA1 is required for normal transcription in Sertoli cells

**Fig. 4:**
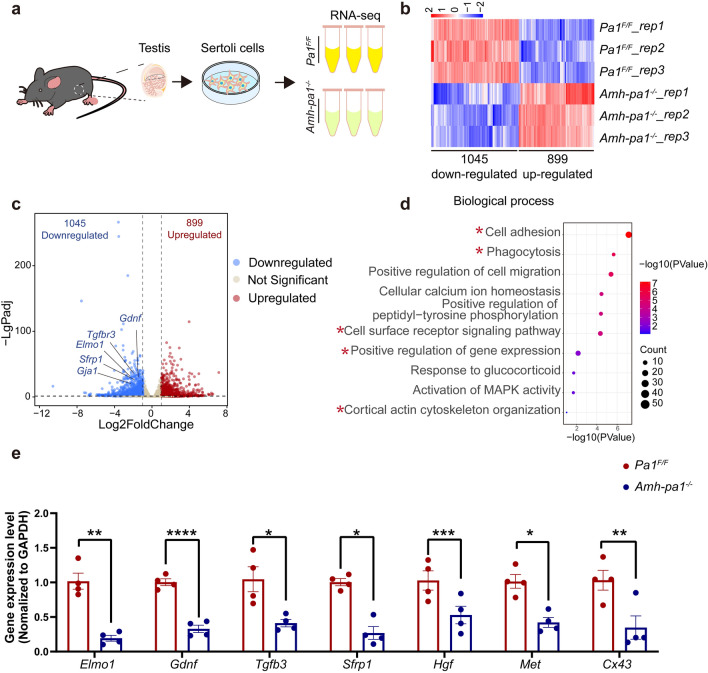
PA1 is required for normal transcription in Sertoli cells. **a** Schematic diagram depicting transcriptome analysis of Sertoli cells. Briefly, Total RNA of wild type and *Pa1*-knockout Sertoli cells from 20-day-old mice testes were collected for the RNA-seq. **b** Heatmaps depicting significantly upregulated and downregulated genes in *Pa1*^*F/F*^ (upper) and *Amh-pa1*^*−/−*^ Sertoli cells (lower). The genes with |log2 Fold change|≥ 1 and q < 0.05 were determined to generate the heatmap. **c** Volcano plot showing DEGs in *Pa1*^*F/F*^ and *Amh-pa1*^*−/−*^ Sertoli cells. The significant changed downregulated genes associated with Sertoli functions were labeled with gene name. **d** GO-Biological process analysis of 1045 downregulated genes in *Amh-pa1*^*−/−*^ Sertoli cells. Biological process of cell adhesion, phagocytosis, positive regulation of gene expression, cell surface receptor signaling pathway, and cortical actin cytoskeleton organization were labeled with asterisks. **e** RT-qPCR of DEGs identified in the volcano plot including *Elmo1*, *Gdnf*, *Tgfb3*, *Sfrp1*, *Hgf*, *Met* and *Cx43* relative to *Gapdh* in *Pa1*^*F/F*^ and *Amh-pa1*^*−/−*^ Sertoli cells. (n = 4). Data are presented as mean ± SEM. *p < 0.05, **p < 0.01, ***p < 0.001 and ****p < 0.0001

Based on the nuclear localization of PA1 in Sertoli cells, we speculated that PA1 may exert its functions by affecting downstream gene expression. Hence, we performed RNA-seq using the total RNA from purified Sertoli cells that were obtained from *Pa1*^*F/F*^ and *Amh-pa1*^*−/−*^ testes from 20-day-old mice (Fig. 4a). Transcriptome analysis revealed that 1045 genes were downregulated, and 899 genes were upregulated (Fig. 4b and c) in *Pa1*-knockout Sertoli cells in contrast to the control Sertoli cells. The overall effect of PA1 in mouse Sertoli cells seems to promote the transcription of downstream genes. Importantly, GO analysis of differentially expressed genes (DEGs) found that the downregulated genes were associated with cell adhesion, phagocytosis, positive regulation of gene expression, and actin cytoskeletal organization (Fig. 4d), whereas upregulated genes were relevant to cell cycle and DNA replication processes (Additional file 8: Fig. S6b, c). Most of the downregulated genes were associated with membrane integrity and function and involved in actin filament binding (Additional file 7: Fig. S5a–c), whereas the upregulated genes were related to chromosomal activities (Additional file 8: Fig. S6a) and associated with calcium ion binding and protein binding (Additional file 8: Fig. S6d).

Within this subset of genes, many of the downregulated genes are reported to play important roles in Sertoli cells, including *Elmo1*, *Gdnf*, and *Sfrp1* (Fig. 4c and e) [32–34]. One notable gene, secreted Frizzled-related protein 1 (sFRP1), is highly expressed in both germ cells and Sertoli cells and has been proved to regulate spermatid adhesion in the testis. In our study, decreased sFRP1 could explain the abnormal loss and exfoliation of germ cells within the seminiferous tubules of the *Amh-pa1*^*−/−*^ testis [34]. Hepatocyte growth factor (HGF) and its receptor Met are reported to be involved during BTB formation, strongly indicating that the compromised integrity of the BTB could partially be due to reduced HGF and Met [35, 36]. In addition, ELMO1 is necessary for the phagocytosis functioning of Sertoli cells, and GDNF is an essential secreted factor of Sertoli cells for spermatogonia self-renewal, corresponding to the aforementioned phenotype. Among the DEGs, we also noticed significantly decreased expression of *Tgfb3*, *Tgfbr3* and others genes related to the TGF-β signaling pathway, implying the importance of PA1 in the regulation of the TGF-β signaling pathway in Sertoli cells. Intriguingly, *Cx43,* which is indispensable for normal Sertoli function, was also significantly decreased, further indicating the dysfunction of *Amh-pa1*^*−/−*^ Sertoli cells. Taken together, transcriptome analyses revealed several key genes associated with normal Sertoli cell functions were differentially expressed when compared *Amh-pa1*^*−/−*^ with wild-type Sertoli cells, indicating PA1 plays critical roles in the transcription regulation of Sertoli cells.

### PA1-associated genes in mouse Sertoli cells

**Fig. 5:**
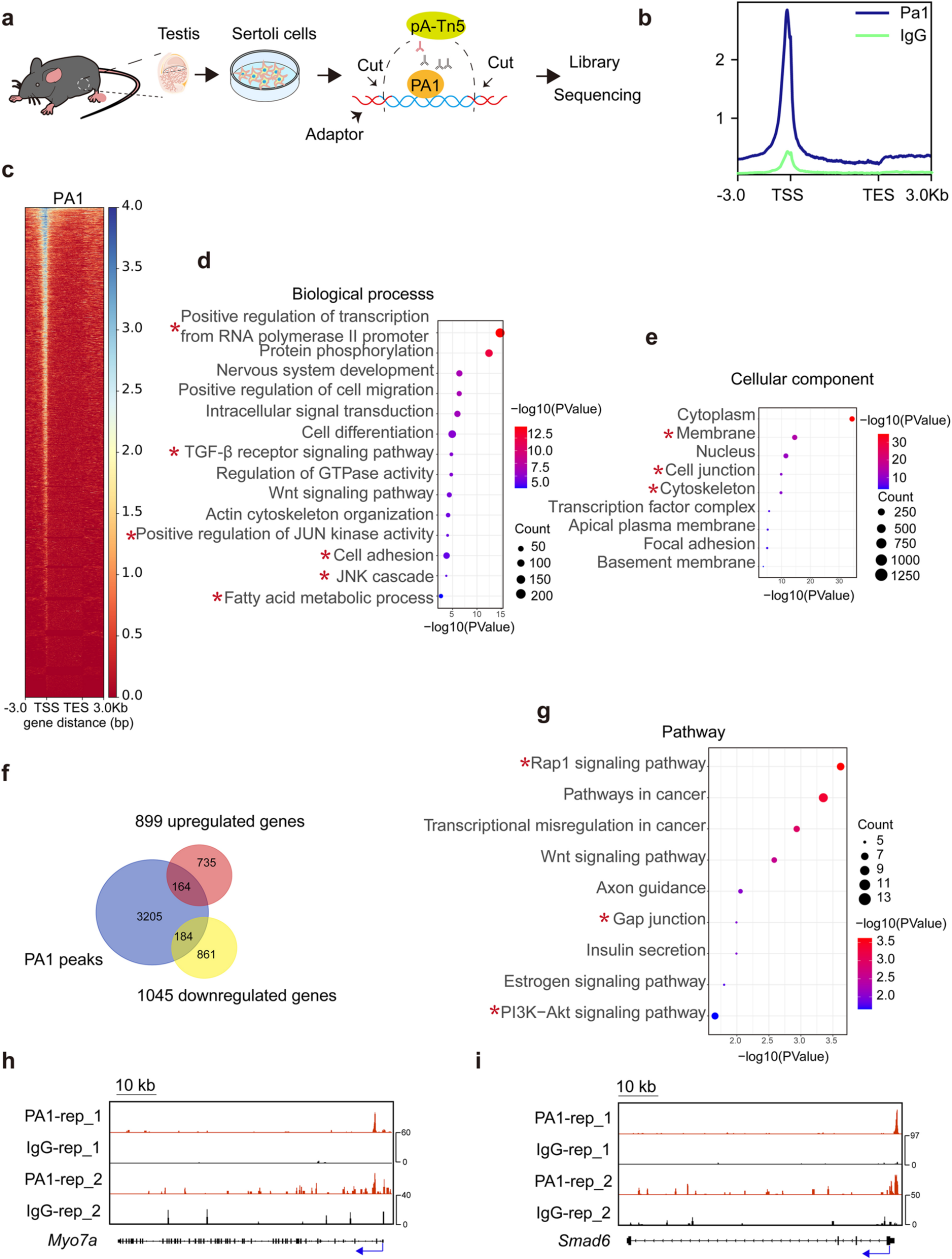
PA1-bound targets in mouse Sertoli cells. **a** Schematic diagram depicting the mechanism of Cut-Tag experiments in the Sertoli cells. Briefly, Sertoli cells from 20-day-old WT mice testes were collected to perform the Cut Tag experiments. Cells were sequentially incubated with anti-PA1 antibody, secondary antibody and pA-Tn5 to specifically cut the PA1-bound DNA regions and add the adapter to the end of DNA fragments. The DNA fragments were next prepared for library and sequencing. **b** Metaplot showing the Cut-Tag signals of PA1 (blue line) and IgG (green line) in Sertoli cells. **c** Heatmap of the binding sites (3553 genes) of PA1 at the positions − 3.0 kb upstream to + 3.0 kb downstream relative to transcription start site (TSS) and transcription end sites (TES). **d** GO-Biological process analysis of PA1 bound genes. Biological processes of positive regulation of transcription from RNA polymerase II promoter, TGF-β receptor signaling pathway, positive regulation of JUN kinase activity, cell adhesion, JUK cascade and fatty acid metabolic process were labeled with asterisks. **e** GO-Cellular component analysis of PA1 bound genes. Cellular components of membrane, cell junction and actin cytoskeleton were labeled with asterisks. **f** Venn diagrams displaying the overlap of downregulated genes and upregulated genes with PA1 bound genes. **g** KEGG pathway analysis of the 184 genes found in both 1045 downregulated genes in *Amh-pa1*^*−/−*^ Sertoli cells and PA1 bound genes. **h** Genomic view of PA1 (Red) and control IgG (Black) at *Myo7a* in wild-type Sertoli cells. Blue arrows indicated transcription start site (TSS). **i** Genomic view of PA1 (Red) and control IgG (Black) at *Smad6* in wild-type Sertoli cells. Blue arrows indicated transcription start site (TSS)

To validate potential PA1-associated targets in Sertoli cells, we performed Cut-Tag experiments with purified Sertoli cells obtained from 20-day-old mice using a PA1 antibody previously validated in ChIP experiments (Fig. 5a) [24]. PA1-bound signals were more prominent at promoter regions compared with IgG-bound signals and could be detected at 3553 genes as shown by the Cut-Tag metaplot and heatmap (Fig. 5b and c). To clarify the functions of these genes, we performed GO analysis and terms such as positive regulation of transcription from RNA polymerase II promoter as well as some ubiquitous signaling pathways such as TGF-β signaling pathway, and positive regulation of JUN kinase activity are enriched in the biological process (Fig. 5d). Cell adhesion was also a notable biological process, and cell junction could be found within cellular components (Fig. 5d). Terms associated with actin cytoskeleton, such as actin cytoskeleton organization and actin binding, were included in results as well (Fig. 5d and Fig. 5e).

Among the DEGs, 184 downregulated genes overlapped with PA1 bound genes while 164 upregulated genes were enriched for PA1 bound genes (Fig. 5f). Previously identified downregulated genes, such as *Gdnf*, *Elmo1*, *Hgf*, *Met* and *Tgfbr3*, were included in this cluster of 184 downregulated genes, further confirming the importance of PA1 in gene regulation. Similar to the results of the transcriptome analysis, GO analysis of the 184 downregulated genes uncovered associations with cell adhesion and actin cytoskeleton organization and enriched pathways included those of gap junctions (Fig. 5g and Additional file 9: Fig. S7a). With actin cytoskeleton binding, MYO7A is known to be an actin binding protein and to be associated with ES in the testis, and we observed strong signals around the *Myo7a* promoter (Fig. 5h). Besides, TGF-β pathways were also significantly enriched in the GO analysis, and we identified strong signals at the promoter of *Smad6,* which is a critical component of the TGF-β signaling pathway. These findings suggest an important role of PA1 in the TGF-β signaling pathway (Fig. 5i and Additional file 9: Fig. S7a), and overall, PA1 might play a prominent role in regulating gene expression by acting on the promoters of target genes.

### PA1 cooperates with JUN to regulate downstream gene expression

**Fig. 6:**
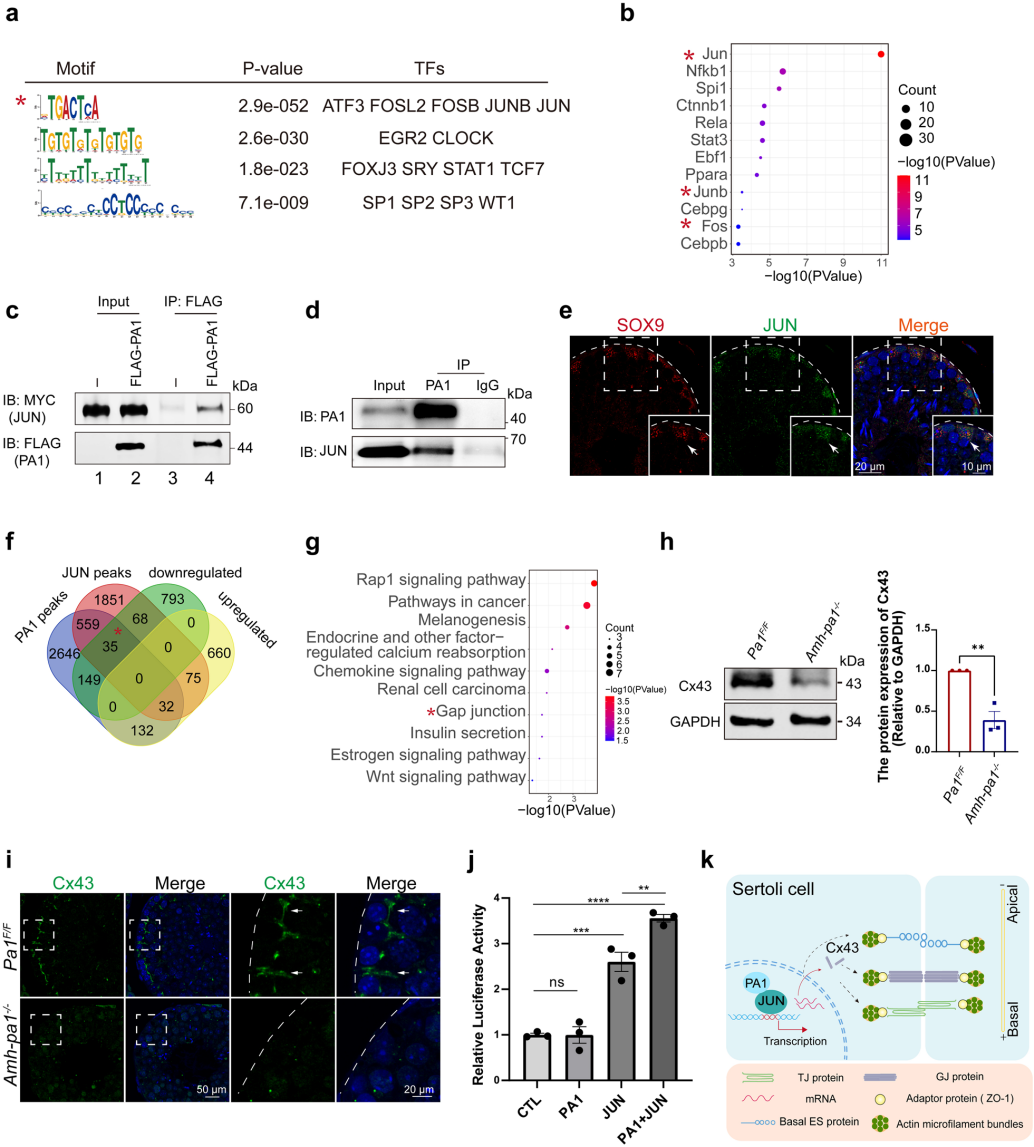
PA1 together with JUN regulates downstream gene expression. **a** PA1 bound motifs identified by MEME in Sertoli cells. The motif sequence is shown in the left column, the P value is shown in middle column and the corresponding transcription factors (TFs) are shown in the right column. The most significant motif sequence was labeled with asterisk. **b** Bubble plot of the upstream transcription factor analysis of 1045 downregulated genes in *Amh-pa1*^*−/−*^ Sertoli cells. The components of AP-1 complex were labeled with asterisks. **c** PA1 was shown to be able to interact with JUN in TM4 Sertoli cells. Lane 1 indicated the input of the *pCS2-Myc-Jun* transfected TM4 cells. Lane 2 represented the input of the TM4 cells co*-*transfected with *pCS2-Myc-Jun* and *pRK-Flag-Pa1*. Lane 3 are the immunoprecipitated products of *pCS2-Myc-Jun* transfected TM4 cells using anti-FLAG antibody*.* Lane 4 are the immunoprecipitated products of the TM4 cells co*-*transfected with *pCS2-Myc-Jun* and *pRK-Flag-Pa1* using anti-FLAG antibody. All th*e lanes* were analyzed with anti-MYC and anti-FLAG antibodies in immunoblot experiment. **d** The endogenous PA1 could interact with JUN. TM4 cells in 6-well plate were transfected with 0.5ug *pCS2-Myc-Jun* plasmid and then the proteins were further immunoprecipitated with PA1 antibody and analyzed with anti-PA1 and anti-JUN antibodies in immunoblot experiment. **e** Immunofluorescence images of JUN in adult mouse testis. Seminiferous tubule sections of wild-type testes were co-stained for Sertoli cell marker SOX9 (red) and JUN (green) and the nuclei was stained with DAPI (blue). Arrows indicated positive cells for positive signals for SOX9 and JUN. Dashed boxes showed localization of the enlarged images. Dashed curve indicated basal membrane of seminiferous tubules. **f** Venn diagrams displaying the overlap of PA1 bound genes, JUN bound genes in MCF-7 cells [38] and downregulated and upregulated genes from the transcriptome analysis. **g** KEGG pathway analysis of 35 overlapped genes among PA1 bound genes in Sertoli cells, JUN bound genes in MCF-7 cells and downregulated genes in *Amh-pa1*^*−/−*^ Sertoli cells. Term of gap junction was labeled with asterisk. **h** Immunoblot analysis and statistical analysis of the protein level of Cx43 in *Pa1*^*F/F*^ and *Amh-pa1*^*−/−*^ testis. (n = 3). Data are presented as mean ± SEM. **p < 0.01. **i** Cx43 signal was diminished in *Amh-pa1*^*−/−*^ mouse testis compared with *Pa1*^*F/F*^ mouse testis. Seminiferous tubule sections of *Pa1*^*F/F*^ mice (upper) and *Amh-pa1*^*−/−*^ mice (lower) were stained for Cx43 (green), and the nuclei was stained with DAPI (blue). Dashed boxes showed localization of the enlarged images. Dashed curve indicated basal membrane of seminiferous tubules. Arrows indicate Cx43 signals at the basal compartment of seminiferous tubules. **j** The overexpression of PA1 together with JUN could promote the promoter activity of *Cx43*. Luciferase reporter assays in TM4 cells were transfected with empty *pCS2* vector, *pCS2-Myc-Pa1*, *pCS2-Myc-Jun* or *pCS2-Myc-Pa1 and pCS2-Myc-Jun* plasmids (n = 3). Data are presented as mean ± SEM. **p < 0.01. ***p < 0.001, ****p < 0.001. **k** PA1 is required for the construction and maintenance of the BTB in mouse Sertoli cells. PA1 functions as a cofactor of JUN to regulate the downstream gene expression in the nuclei of Sertoli cells, such as *Cx43*, and participate in maintaining the functional BTB. Moreover, PA1 may also regulate downstream gene expression associated with BTB regulation, such as basal ES, contributing to the integrity of the BTB

Although PA1 appears to be indispensable for normal transcription in Sertoli cells, known DNA-binding domains have not been identified within the PA1 protein. Therefore, PA1 would need to interact with other proteins that possess DNA-binding domains to jointly affect downstream gene expression. To reveal the proteins that potentially interact with PA1, we performed motif analysis of PA1-targeted DNA sequences obtained from Cut-Tag results. Notably, the motif analysis showed that the most prevalent DNA element was -TGACTCA-, which is the typical binding site for the transcription Activation Protein 1 (AP-1) complex, including JUN, FOSB, FOS as well as other transcription factors (Fig. 6a) [37]. Interestingly, upstream analysis of the 1045 downregulated genes found that the most significant transcription factor was JUN (Fig. 6b), strongly supporting the close relationship between JUN and PA1. Supporting studies were done to compare control MEFs with PA1-deleted MEFs, and the identified downregulated genes were then used to perform upstream analysis. These analyses revealed that the most enriched upstream transcription factor was indeed JUN (Additional file 9: Fig. S7b) [24]. To determine whether PA1 could actually interact with JUN, we performed co-immunoprecipitation experiments of PA1 and JUN using the TM4 Sertoli cell line. Results showed that PA1 did indeed interact with JUN (Fig. 6c). However, we did detect a relatively low expression of endogenous JUN in the TM4 Sertoli cells which may compromise the detection of endogenous interaction of PA1 and JUN (Additional file 9: Fig. S7c). Hence, we tried to transfected the TM4 Sertoli cells with a relative low level of *pCS2-Myc-Jun* plasmids and the proteins collected from the transfected cells were immunoprecipitated with the anti-PA1 antibody. With this method, we did detect the strong interaction between PA1 and JUN (Fig. 6d). Next, to determine whether JUN also localizes in Sertoli cells in mouse testis, we performed an immunofluorescence analysis of JUN in mouse testis with a specific marker for Sertoli cells, Sox9. We did find that JUN mainly localized in Sertoli cells (Fig. 6e), further confirming the possible interaction of JUN with PA1 in mouse testis. We also determined that c-Fos and JunB were enriched as upstream transcription factors of the downregulated genes, implying the potential interaction between other AP-1 complex components and PA1.

Next, we compared ChIP-seq data of JUN performed in MCF-7 cells with PA1 Cut-Tag results performed in Sertoli cells [38]. Our comparison revealed that the 626 overlapped genes were observed with JUN bound genes in MCF-7 cells and PA1 bound genes in Sertoli cells (Additional file 9: Fig. S7d). GO analysis of these 626 genes identified relationships with adherens junction, gap junction, actin binding and actin filament binding (Additional file 9: Fig. S7e–g). Further analysis with our transcriptome results uncovered that 35 genes coexisted within downregulated genes, JUN signals and PA1 signal clusters (Fig. 6f). GO analysis of these 35 genes found that gap junction was still one of the most significant terms (Fig. 6g). Among the genes related to gap junction, it has been reported that a functional relationship between JUN and Sox8 or Sox9 co-regulates the expression of Cx43 via the recruitment of JUN to the mouse *Cx43* promoter [39, 40]. Our transcriptome results did indeed reveal a significant decrease of *Cx43* in *Amh-pa1*^*−/−*^ Sertoli cells, as the RT-qPCR results confirmed (Fig. 4e). To further confirm the regulation of PA1 on Cx43, we conducted immunofluorescence and immunoblot analyses on control and *Amh-pa1*^*−/−*^ mice testes. The immunoblot experiment found a significant decrease of Cx43 in the *Amh-pa1*^*−/−*^ testis(Fig. 6h, i). Notably, we could hardly detect signals of Cx43 in the *Amh-pa1*^*−/−*^ testis whereas in the control mice, Cx43 localized at the basal compartment of seminiferous tubules (Fig. 6i). Further dual-luciferases experiments were performed to testify the transcription co-regulation of PA1 and JUN on the promoter of *Cx43*. The results showed that solely transfection of PA1 could not increase the transcriptional activity of *Cx43* promoter while the overexpression of JUN could promote the activity of *Cx43* promoter which is consistent with the previous published studies. The overexpression of PA1 as well as JUN did significantly enhance the promoter activity of Cx43 compared with the control group and the group solely transfected with JUN, suggesting that the transcription co-regulation of PA1 and JUN in TM4 cells (Fig. 6j). Hence, we conclude that PA1 may cooperate with JUN to regulate the transcription of *Cx43* and probably other BTB-related genes in Sertoli cells.

## Discussion

In the present study, we revealed a novel mechanism underlying BTB integrity in a mouse model with specific knockout of *Pa1* in Sertoli cells. Depletion of PA1 in Sertoli cells resulted in aberrant spermiogenesis through disorganization of the actin cytoskeleton and BTB components in Sertoli cells, ultimately leading to the gradual loss of different types of germ cells. Transcriptome and Cut-Tag experiments revealed that PA1 could cooperate with JUN to regulate downstream transcription of genes, such as *Cx43,* that are indispensable for spermatogenesis. Overall, our study reveals that PA1 is required for spermatogenesis in Sertoli cells and plays essential roles, such as maintaining the BTB and regulating actin cytoskeleton organization (Fig. 6k).

The *Amh-pa1*^*−/−*^ mice presented significantly decreased testis volume and weight, and we could contribute this result to the dramatic loss of germ cells. Using different germ cell markers include the PCNA, DDX4 and PLZF, we did find the number of all stages of germ cells were significantly reduced. Further TUNEL assay verified more remarkable apoptotic germ cells in the *Amh-pa1*^*−/−*^ seminiferous tubules, which could account for the smaller testis. Besides that, the smaller testis could also be attributed to the reduced growth factors such as GDNF detected by the RNA-seq, one of the important secreted factors by Sertoli cells, essential for the self-renew of SPGs, which compromised the proliferation of SPGs.

Among the defects of *Amh-pa1*^*−/−*^ mice, we mainly focused on the role of PA1 in the regulation of BTB structure, observing a dysfunctional and disorganized BTB in *Amh-pa1*^*−/−*^ testis. Furthermore, we found the classical gap junction protein, Cx43, which has been revealed to be indispensable for normal spermatogenesis in mouse testis, could be the direct target of PA1. Some similarities could be found between the *Cx43*-Sertoli cell-specific knockout mice and *Amh-pa1*^*−/*−^ mice. First, in *Cx43*-Sertoli cell-specific knockout mice, spermatogenesis was arrested at the spermatogonia stage and some tubules were empty, whereas in the adult *Amh-pa1*^*−/*−^ mice [41], we found that germ cells were completely absent in some tubules and undifferentiated spermatogonia were significantly reduced. Second, the disorganization of the BTB could be located to the basal compartment of the seminiferous tubules in these two types of mice. In *Cx43* Sertoli cell-specific knockout mice, BTB components such as ZO-1, β-Catenin and Occludin were diffused and presented as clusters and clumps among the Sertoli cells [42], which resemble the distribution of these proteins in *Amh-pa1*^*−/−*^ testis. Meanwhile, the expression of ZO-1 was significantly decreased in the testis of *Cx43* Sertoli cell-specific knockout mice as well as the *Amh-pa1*^*−/−*^ mice in contrast to wild-type mice, implying the indirect regulation of PA1 on ZO-1 expression [43]. In addition, the disordered F-actin arrangement in the basal seminiferous tubules also contributed to the disruption of the BTB barrier at Sertoli-Sertoli junctions.

The AP-1 complex has been demonstrated to play essential roles in diverse physiological processes, including cell proliferation, cell migration, apoptosis, and transformation in different organs [37, 44–47]. Previous studies have reported homozygous *Jund*^*−/−*^ mutant male mice showed defective spermatogenesis and decreased fertility, and the knockout of *c-Fos* also blocked normal spermatogenesis [48, 49]. JUN is an important component of the AP-1 complex, which is known for transcription activation, and also plays essentials roles in different biological processes, including the development of heart and liver, tissue scarring and others [50–52]. In fact, *Jun*^*−/−*^ mice have demonstrated arrested embryonic development at E13 due to the failure of organ development [51]. Little is known about the function of JUN in testis. In TM4 Sertoli cells, JUN was found to cooperate with other transcription factors including Sox9, SF-1 and Nur77, affecting the expression of Cx43 [39, 40]. As a component of the AP-1 complex, JUN was unexpectedly expressed primarily in the nuclei of Sertoli cells, corresponding to the dominant nuclear localization of PA1 in Sertoli cells. The specific knockout of *Pa1* in Sertoli cells influenced a subset of genes, which could be regulated by JUN. Additionally, Cut-Tag results revealed the common binding motif between JUN and PA1, suggesting the interaction between these two proteins in Sertoli cells. Besides testis, in Schwann cells, the absence of JUN also resulted in decreased GDNF which was downregulated in *Amh-pa1*^*−/−*^ Sertoli cells, interfering with axonal regeneration [53]. In the hepatocellular carcinoma cell line Hepa 1–6, overexpression of JUN together with FOS could enhance the activity of MET, which also seems to be a downstream target of PA1 [54]. Overall, the interaction of PA1 and JUN transcription regulation might be conserved in different biological processes and deserves to be further explored. Besides JUN, we also detected that FOS, JUNB, ATF and other AP-1 components share some common binding targets with PA1 and act as upstream transcription factors for downregulated genes in *Amh-pa1*^*−/−*^ Sertoli cells, further suggesting that PA1 may be involved in other versatile functions performed by AP-1 complex components.

Evolutionarily, the protein sequences of PA1 are conserved among different species [55]. We did indeed find similar expression patterns of PA1 between mouse and human testicular Sertoli cells, indicating a possible conserved function. During the male embryonic development, the somatic progenitors in fetal gonads differentiate toward the Sertoli cells. PA1 depletion or mutation in somatic progenitors are not supposed to affect embryonic development, which may lead to azoospermia in adulthood. In addition to JUN, PA1 may also interact with some other Sertoli cell-specific cofactors, and some mutation(s) in this area may not disturb its function in embryonic development, but affect male fertility. Besides, due to the potential vital role of PA1 as a nuclear regulator of Sertoli cells, it might be involved with general abnormalities in Sertoli cells caused by other factors. Similar to PA1, JUN also showed a remarkable localization to the nuclei of Sertoli cells in mouse testis, and previous research has reported the localization of JUN to human Sertoli cells, further implying the conserved cooperation between JUN and PA1 in human Sertoli cells [56]. Further studies will be performed to confirm the possibility that the cooperation between PA1 and JUN regulating the expression of Cx43 is also conserved in human Sertoli cells.

## Conclusions

In summary, our study is the first to identify a nuclear transcriptional cofactor highly expressed in Sertoli cells that can interact with AP-1 complex components to influence the expression of downstream genes involved in normal Sertoli cell functions. These genes are crucial for maintaining the ordered arrangement of the BTB, organizing the actin cytoskeleton, and ultimately preserving male fertility. Thus, this study provides a new clinical diagnostic or even therapeutic target for individuals with azoospermia.

## Materials and methods

### Animals

*Pa1*^*F/F*^ mice were gifts from Dr. Kai Ge (National Institutes of Health). *Pa1*^*F/F*^ mice were bred with *Amh-cre* mice to generate *Amh-cre*, *Pa1*^*F/*+^ (F1). *Amh-cre*, *Pa1*^*F/*+^ were mated with *Pa1*^*F/F*^ mice to generate *Amh-cre*, *Pa1*^*F/F*^ (*Amh-pa1*^*−/−*^) mice (F2). All mice are kept in a specific pathogen-free (SPF) environment. The primers used for genotyping were listed in Additional file 1: Table S1.

### Plasmids and antibodies

Mouse *Pa1* and *Jun* amplified from mouse testis cDNA were cloned into the *pRK-flag* and *pCS2-myc vector*, respectively, according the protocol of Clon Express Kit (C115, Vazyme). Rabbit anti-PA1 antibody (ABE1863) was used for western blot and Cut-Tag experiments, rabbit anti-PA1 (NBP1-89792) was used for immunofluorescence and immunohistochemistry. Rabbit anti-SYCP3 (ab15093) and mouse anti-γH2AX (05–636) were used for immunofluorescence. Rabbit anti-DDX4 antibody (ab13840), mouse anti-PCNA (ab29) and goat anti-PLZF (AF2944) were used for immunofluorescence. Rabbit anti-WT1 (ab89901) and mouse anti-WT1 (NB110-60011) were used for immunofluorescence. Rabbit anti-CLAUDIN11 antibody (ab53041) was used for immunofluorescence and western blot. Rabbit anti-OCCLUDIN (711500) antibody, rabbit anti-CTTNB1 (712700) antibody, and rabbit anti-ZO-1 antibody (402200) from Invitrogen was used for immunofluorescence and western blot. Rabbit anti-GJA1 (26980-1-AP) and rabbit anti-GJA1 (A11752) were used for immunofluorescence and western blot experiments. Rabbit anti-JUN (A0246) was used for immunofluorescence experiments. Rabbit anti-MYC (BE2011), mouse anti-MYC (M20002L), and mouse anti-Flag (M20008L) were used for immunoprecipitation and immunoblot experiments. Mouse anti-GAPDH (AC002) antibody and mouse anti-β-TUBULIN (AC021) antibody were purchased from ABclonal. Rabbit IgG (B30011), purchased from Abmart, was used for Cut-Tag experiments. FITC-labeled Phalloidin (40735ES75) purchased from Yeasen was used for immunofluorescence (Shanghai, China). TRITC- or FITC-labeled goat anti-mouse, TRITC- or FITC-labeled goat anti-rabbit, and HRP-conjugated goat anti-rabbit secondary antibodies (Zhong Shan Jin Qiao, Beijing, China) were used for immunohistochemistry and immunofluorescence experiments. Alexa Fluor 680-labeled goat anti-mouse and Alexa Fluor 800-labeled goat anti-rabbit secondary antibodies purchased from Invitrogen were used for western blot.

### Fertility assessment of mice

Fertility experiments were conducted as previously described [16]. 8-week-old male mice were caged with 8-week-old female mice at a ratio of 1:2. The vaginal plugs were checked the next day and the male mice were separated alone for next two days to have a rest. After that, the male mice were further caged with new 8-week-old female mice again. During the whole process, the pregnancy rate and number of litters of at least five female mice with positive vaginal plugs were recorded.

### Isolation and primary culture of Sertoli cell

The isolation of primary Sertoli cell was performed as previous description with some modifications [16]. In brief, the testis of 20-day-old mice were decapsulated and washed with PBS for 3 times. The seminiferous tubules were further digested with 2 mg/ml collagenase IV (Sigma, C5138) in PBS for 15 min at 37 °C, and digested with 1 mg/ml hyaluronidase (Sigma, H3506) and 2 mg/ml collagenase IV and in PBS for 10 min at 37 °C. After that, the tubules were then dispersed through pipetting up and down into small short tubules, and centrifuged at 30×*g* for 3 min at room temperature. The cells were further digested with a mixture of 2 mg/ml hyaluronidase, 2 mg/ml collagenase IV and 0.5 mg/ml DNase I in 1 mg/ml trypsin (Gibco, 25200072) and incubated in 2 ml Eppendorf tubes for 15 min at 37 °C. The digestion was halted using equal volume of F12-DMEM (HyClone, SH30023.01B) including 15% fetal bovine serum (Gibco, 10270). The products were filtered through a 200-mesh filter (Solarbio, YA0961), and washed twice with PBS centrifuged at 600×*g* for 3 min at room temperature. The separated cells were resuspended with F12-DMEM (HyClone, SH30023.01B), 10 μg/ml insulin, 2.5 ng/ml EGF, and 5 μg/ml transferrin and cultured in 35 mm plastic dishes (CORNING, 4141) at 34 °C and 5% CO_2_. After 8 h, the dishes were gently washed with PBS twice to remove the unattached germ cells.

### TM4 Sertoli cell line culture and cell transfection

Mouse TM4 Sertoli cell line from the American Type Culture Collection (ATCC, Manassas, VA, USA) were cultured in 35 mm plastic dishes (CORNING, 4141) with F12-DMEM containing 15% fetal bovine serum (Gibco, 10270) at 37 °C and 5% CO_2_. When the cell density reached 60–80% in 6-well plates (Beijing Labgic Technology, 11110), the cells were further transfected with the constructed plasmid DNA (2 ng) with lippomax (Invitrogen, 11668019) for 8 h followed the protocol. Cells were harvested 2 days post transfection for further experiments.

### Separation of spermatogenic cells

Separation of germ cells was performed using a STA-PUT method with minor modification as previously described [57] with detailed information in the Additional file 2.

### Histological analysis

Testes and caudal epididymides for each genotype of mice were fixed in Bouin’s solution or 4% (m/v) paraformaldehyde (PFA) for 24–48 h, embedded in paraffin, and cut into 5 μm sections. After deparaffinization, the tissue sections were further stained with hematoxylin and eosin (H&E). For Periodic acid-Schiff staining, deparaffinized slides were stained with PAS and hematoxylin followed the protocol in the PAS kit (G1280, Solarbio).

### Transmission electron microscopy

After the mice were anesthetized, 50 ml of fixative solution (2% paraformaldehyde and 2% glutaraldehyde) was perfused into the heart within 1 h, and the testes were then removed and fixed in the fixative solution for 4 h. After washed with 0.1 M PB buffer (80 mM Na_2_HPO_4_ and 25 mM NaH_2_PO_4_) for 3 times, the tissues were immersed in 1% osmium acid for 2 h. Through the acetone gradient dehydration and immersion of acetone: epoxy resin (1:1), the tissues were embedded, and placed at 60 °C for 24 h. The sections (70 nm) were sliced via the Leica EM UC7 ultra-thin microtome, and stained with uranyl acetate and lead citrate. Images were captured and analyzed with JEM-1400 electron microscope and Gatan digital camera.

### Immunoblotting and immunoprecipitation

For immunoblotting, the cells or testes proteins were extracted with cold-RIPA buffer with the addition of protein inhibitor cocktail (Roche Diagnostics, 04693132001), separated via SDS-PAGE and electrotransferred to a nitrocellulose membrane. The membrane was further blocked with 5% milk and incubated in corresponding primary antibody. In the next day, the membrane was further incubated with Alexa Fluor 800 or 680-labeled goat anti-rabbit or Alexa Fluor 800 or 680-labeled goat anti-mouse secondary antibodies. Finally, the ODYSSEY System (LI-COR Biosciences, Lincoln, NE, RRID:SCR_014579) was used to scan the membrane.

For Immunoprecipitation, the transfected cells proteins were extracted with the TAP buffer (50 mM HEPES–KOH, pH7.5, 2 mM EDTA, 100 mM KCl, 10 mM NaF, 10% glycerol, 0.25 mM Na_3_VO_4_, 0.1% NP-40, 50 mM β-glycerolphosphate) with the addition of protein inhibitor cocktail, and further incubated with relevant primary antibody at 4 ℃ overnight. Protein A-Sepharose beads (GE, 17-1279-03) were prepared and added to the proteins at 4 ℃ for 2 h. Immunoprecipitated product were washed 3 times with TAP buffer with centrifuged at 845×*g* for 5 min at 4 ℃, and the pellets were eluted with loading buffer containing 1% SDS, heated for 10 min at 95 ℃ and examined by immunoblotting.

### Immunofluorescence, TUNEL assay and immunohistochemistry

Immunofluorescence and immunohistochemistry were performed as previous published articles [16] with detailed information in the Additional file 2. For TUNEL assay, after the secondary antibody incubation, enzyme and labeling solutions were mixed followed the manufacturer’s protocol (11684817910, Roche). The mixture was then used to incubate the sections at 37 °C for 1.5 h. And images were acquired using an LSM 780/710 microscope (Zeiss, Germany) or SP8 microscope (Leica, Germany).

### In vivo BTB integrity analysis assay

The integrity of the BTB was detected as Chen et al. reported [58]. In brief, unilateral testis of anesthetized mice were exposed and fine-pointed needle was inserted to the interstitium of testis followed the injection of 20 ul of 10 mg/ml Biotin (F3272; Sigma). After 30 min, the testes were removed and embedded in optimal cutting temperature compound (OCT; Tissue-Tek, 4583) and sliced into 7 mm sections using a slicer-cryostat. The sections were washed using PBS and then incubated in a 1:200 mixture of FITC-labeled-Streptavidin (Zhong Shan Jin Qiao, Beijing, China) for 2 h at 37 °C. DAPI was used to stain the nuclei. Results were taken as previous described.

### Cut-Tag experiments and data analysis

Cut-Tag experiments and data analysis were performed followed the published articles [59, 60] with minor modification. The detailed information was presented in the Additional file 2. Data from Cut-tag were aligned to the reference mm39 genome with Bowtie v2.2.3 using default settings and the duplicates and mitochondrial genome were removed by Samtools. MACS2 was used to call for the peaks using default parameters. With bamCoverage from deepTools, the aligned files (Binary Alignment Map [BAM]) were transformed to normalized coverage files (bigWig) for the visualization of specific signals. BamCoverage from deepTools was used for normalization. Images of the signals were captured using the Integrative Genomics Viewer (IGV). Heatmaps were generated with ComputeMatrix from deepTools. The motif analysis was performed using MEME software (https://meme-suite.org).

### Dual-luciferase reporter assay

The *Gja1* promoter (− 2000 to + 36) was cloned into the *pGL3-basic* plasmid (Tsingke, Beijing). TM4 cells seeded in 24-well-plate were transfected using lippomax (Invitrogen, 11668019) with 20 ng plasmids containing Renilla luciferase, 200 ng of plasmids encoding firefly luciferase reporters and empty *pCS2* vector, *pCS2-pa1*, *pCS2-jun or pCS2-pa1* with *pCS2-jun* plasmids. The luciferase activity was measured using the Dual-Lumi™ Luciferase Reporter Gene Assay Kit (RG088S).

### RNA-seq library construction and data analysis

Total RNA of Sertoli cells from 20-day-old mice testes was extracted with Trizol (Invitrogen). NEBNext® Ultra™ RNA Library Prep Kit from Illumina® (#E7530L, NEB, USA) was used to construct the libraries. Sequencing was carried out with Illumina HiSeq 2000. The genome index was built with Bowtie2 v2.2.3, and the Clean Data was aligned to the reference genome, Mus_musculus.GRCm38.90.chr, with HISAT2 v2.1.0. HTSeq v0.6.0 was used to count the reads number of each gene in each sample. FPKM (Fragments Per Kilobase Millon Mapped Reads) was calculated to estimate the expression level of genes in each sample. Differential gene expression was analyzed with with DEGseq v1.18.0 between the samples in biological replicates. The genes with |log2 Fold change|≥ 1 and q (padj) < 0.05 were determined as differentially expressed genes. GO analyses were performed using DAVID27. Upstream analysis was done using Metascape (https://metascape.org).

### Statistical analysis

The statistical analysis was conducted with GraphPad PRISM 9, and the results are expressed as mean ± SEM. All the experiments were repeated at least three times independently. The statistical significance was examined using one-way ANOVA with Dunnett’s multiple comparison test and Sidak multiple comparison test or the two tailed Student’s t-test. The significance of data presented as P < 0.05 (*), 0.01(**), 0.001(***) and 0.0001(****).

## Supplementary Information

**Additional file 1.** The primers used in this study including Table S1 and Table S2.

**Additional file 2.** The detailed supplementary methods and materials mentioned in the article.

**Additional file 3: Fig. S1.** The expression pattern of PA1 in human and mouse. a) PA1 protein levels gradually increased during the development of testis. The expression pattern of PA1 in different developmental stages of murine testes (Day 7, Day 14, Day 21, Day 28, Day 35, Day 56) using Western Blot. Statistical analysis of the expression level of PA1 during the development of testis was showed in the lower panel. b) Semi RT-PCR results showed *Pa1* was ubiquitously expressed in different mouse organs but especially enriched in the testis. *β-actin* was used as the loading control. c) Semi RT-PCR results showed *Pa1* levels gradually increased in mouse testis with the development of testis. *β-actin* was used as the loading control. d) PA1 is localized in Sertoli cells in mouse testis during the whole spermatogenic cycle while the expression level of PA1 in different stages varies. Testes sections from adult wild-type mice (8-week-old) were stained for PA1 using immunohistochemistry as indicated. Arrows indicate positive cells for PA1 expression. e) PA1 was expressed in different testicular cells but especially abundant in Sertoli cells. Immunoblotting of PA1 was performed in Sertoli cells, SC; spermatogonium, SPG; spermatocytes, SPC; round spermatids, rST; elongated and elongating spermatids, eST.

**Additional file 4: Fig. S2.** Generation of *Amh-pa1*^*−/−*^ mice. a) Schematic diagram for the generation of *Amh-pa1*^*−/−*^ mice. Exon 1 and exon 2 (blue) of *Pa1* were flanked by loxp sites (yellow) in *Pa1*^*F/F*^ mice. These exons were specifically deleted in the Sertoli cells in *Amh-pa1*^*−/−*^ mice. b) *Amh-pa1*^*−/−*^mice genotyping. The 250 bp band indicated floxed *Pa1,* the 200 bp band indicated *Pa1*, and the 400 bp band indicated *Amh-cre*. c) PA1 was specifically knocked out in testicular Sertoli cells of 5-week-old mice. Seminiferous tubule sections of *Pa1*^*F/F*^ and *Amh-pa1*^*−/−*^ testes were co-stained for WT1(green) and PA1 (red). The nuclei were stained with DAPI (blue). Solid boxes showed localization of the enlarged images. Dashed circle indicated basal membrane of seminiferous tubules. Arrows indicated the nuclei of Sertoli cells. d) Body weights of 8-week-old *Pa1*^*F/F*^ mice and *Amh-pa1*^*−/−*^ male mice (n = 5 independent experiments) were similar. Data are presented as mean ± SEM. e) Testis weights of *Amh-pa1*^*−/−*^ mice were significantly lower than those of *Pa1*^*F/F*^ mice (n = 5 independent experiments). Data are presented as mean ± SEM. ****p < 0.0001.

**Additional file 5: Fig. S3.** Disorganized basal actin cytoskeleton is detected in *Amh-pa1*^*−/−*^ mice testis. F-actin at the basal compartment of seminiferous tubules was observed to be perturbed and disorganized in adult *Amh-pa1*^*−/−*^ testes. Arrows indicate F-actin bundles. Immunofluorescence using FITC-labeled phalloidin (green) was performed on the frozen testis sections of *Pa1*^*F/F*^ mice (upper panels) and *Amh-pa1*^*−/−*^ mice (lower panels). Nuclei were stained with DAPI (blue). Dashed boxes showed the localization of the enlarged images. Dashed curve indicated basal membrane of seminiferous tubules.

**Additional file 6: Fig. S4.** *Amh-pa1*^*−/−*^ mice show massive germ cell loss. a) Most of the residual cells in the empty tubules of *Amh-pa1*^*−/−*^ testis were identified as Sertoli cells. Immunofluorescence analysis using anti-WT1 (green) was conducted on the sections of testis from *Pa1*^*F/F*^ mice (upper panels) and *Amh-pa1*^*−/−*^ mice (lower panels). Nuclei were stained with DAPI (blue). Dashed curve indicated basal membrane of seminiferous tubules. b) PCNA immunofluorescence analysis of seminiferous tubules showed that 8-week-old *Amh-pa1*^*−/−*^ mice had decreased numbers of spermatocytes and spermatogonia compared with 8-week-old *Pa1*^*F/F*^ mice. Seminiferous tubule sections of *Pa1*^*F/F*^ and *Amh-pa1*^*−/−*^ testes were stained for PCNA (red), and the nuclei was stained with DAPI (blue). Dashed boxes showed the localization of the enlarged images. Dashed curve indicated basal membrane of seminiferous tubules. Data are presented as mean ± SEM. ***p < 0.001. c) DDX4 immunofluorescence analysis of germ cells showed that 8-week-old *Amh-pa1-/-* mice had decreased numbers of germ cells compared with 8-week-old *Pa1*^*F/F*^ mice. Seminiferous tubule sections of *Pa1*^*F/F*^ and *Amh-pa1*^*−/−*^ testes were stained for DDX4 (red), and the nuclei was stained with DAPI (blue). Dashed boxes showed the localization of the enlarged images. Dashed curve indicated basal membrane of seminiferous tubules. Data are presented as mean ± SEM. **p < 0.01. d) PLZF immunofluorescence analysis of germ cells showed that 8-week-old *Amh-pa1*^*−/−*^ mice had decreased numbers of undifferentiated spermatogonium compared with 8-week-old *Pa1*^*F/F*^ mice. Seminiferous tubule sections of *Pa1*^*F/F*^ and *Amh-pa1*^*−/−*^ testes were stained for PLZF (red), and the nuclei was stained with DAPI (blue). Dashed boxes showed the localization of the enlarged images. Dashed curve indicated basal membrane of seminiferous tubules. Arrows indicated PLZF positive cells. e) PCNA positive cell number per tubule was significantly decreased *Amh-pa1*^*−/−*^ (27.34 ± 1.319) mice testes compared with *Pa1*^*F/F*^ mice (62.72 ± 3.167). (n = 3 independent experiments). Data are presented as mean ± SEM. ***p < 0.001. f) DDX4 positive cell number per tubule was significantly decreased in *Amh-pa1*^*−/−*^ (50.39 ± 2.342) mice compared with *Pa1*^*F/F*^ mice (122.9 ± 12.20). (n = 3 independent experiments). Data are presented as mean ± SEM. **p < 0.01. g) PLZF positive cell number per tubule was significantly decreased *Amh-pa1*^*−/−*^ (0.5789 ± 0.1919) mice testes compared with *Pa1*^*F/F*^ mice (2.763 ± 0.2321). (n = 3 independent experiments). Data are presented as mean ± SEM. ***p < 0.001. h) Homologous chromosome synapsis, pairing, or segregation were normal undergoing in *Amh-pa1*^*−/−*^ spermatocytes. Spermatocytes of *Pa1*^*F/F*^ and *Amh-pa1*^*−/−*^ mice were stained with SYCP3 (green) and γH2AX (red) antibodies.

**Additional file 7: Fig. S5.** 5 GO analysis of downregulated genes in *Amh-pa1*^*−/−*^ Sertoli cells. a) GO-Molecular function analysis of 1045 downregulated genes in *Amh-pa1*^*−/−*^ Sertoli cells. Molecular function of protein binding, actin filament binding and actin binding were labeled by asterisks. b) GO-Cellular component analysis of 1045 downregulated genes in *Amh-pa1*^*−/−*^ Sertoli cells. Cellular component of membrane, cell junction and actin cytoskeleton were labeled with asterisks. c) KEGG pathway analysis of 1045 downregulated genes in *Amh-pa1*^*−/−*^ Sertoli cells. KEGG pathways of phagosome, cell adhesion molecules, regulation of actin cytoskeleton and focal adhesion were labeled with asterisks.

**Additional file 8: Fig. S6.** GO analysis of upregulated genes in *Amh-pa1*^*−/−*^ Sertoli cells. a) GO-Cellular component analysis of 899 upregulated genes in *Amh-pa1*^*−/−*^ Sertoli cells. Cellular component of chromosome was labeled with asterisks. b) KEGG pathway analysis of 899 upregulated genes in *Amh-pa1*^*−/−*^ Sertoli cells. KEGG pathways of cell cycle was labeled with asterisks. c) GO-Biological process analysis of 899 upregulated genes in *Amh-pa1*^*−/−*^ Sertoli cells. Biological process of cell cycle was labeled with asterisks. d) GO-Molecular function analysis of 899 upregulated genes in *Amh-pa1*^*−/−*^ Sertoli cells. Molecular function of calcium ion binding, and actin binding were labeled by asterisks.

**Additional file 9: Fig. S7.** PA1 together with JUN regulates downstream gene expression. a) GO-Biological process analysis of the 184 overlapped genes identified in both downregulated genes of *Amh-pa1*^*−/−*^ Sertoli cells and PA1 bound genes. Biological process of actin cytoskeleton organization, cell adhesion and transforming growth factor beta receptor signaling pathway were labeled with asterisks. b) Upstream transcription factor analysis of downregulated genes in *Pa1-*knockout MEFs. The identified top 1 transcription factor JUN was labeled with asterisk. c) The endogenous co-immunoprecipitation results of PA1 and JUN in TM4 cells. The proteins of TM4 cells were collected and immunoprecipitated using anti-PA1 antibody and the products were further used for immunoblot analysis with anti-PA1 and anti-JUN antibodies. The bands for PA1 and JUN were labeled with the arrows. d) Venn diagram showing the overlap of PA1 bound genes in Sertoli cells with JUN bound genes in MCF-7 cells [43]. e) GO-Molecular function analysis of the 626 overlapped genes identified in both PA1 bound genes in Sertoli cells and JUN bound genes in MCF-7 cells [43]. Molecular function of protein binding, actin binding and actin filament binding were labeled with asterisks. f) GO-Biological process analysis of the 626 overlapped genes identified in both PA1 bound genes in Sertoli cells and JUN bound genes in MCF-7 cells [43]. Biological process of cell adhesion was labeled with asterisks. g) KEGG pathway analysis of the 626 overlapped genes identified in both PA1 bound genes in Sertoli cells and JUN bound genes in MCF-7 cells. KEGG pathway of adherens junction and gap junction were labeled with asterisks.

## Data Availability

All the data supporting the results of this research could be obtained from this article and its Additional file 2. All raw high-throughput sequencing data have been uploaded to the GEO, and the GEO accession number is GSE188337.
