# Supplementary material for: PA1 participates in the maintenance of blood–testis barrier integrity via cooperation with JUN in the Sertoli cells of mice
Source: Cell Biosci. 2022 Apr 4;12:41. doi: 10.1186/s13578-022-00773-y (PMC8981650; doi:10.1186/s13578-022-00773-y)
Supplement: Supplementary file 1 — Additional file 1. The primers used in this study including Table S1 and Table S2. [file 13578_2022_773_MOESM1_ESM.docx]

**Table S1**

| **Genotype** |  |  |
| --- | --- | --- |
| **Gene** | **Forward primer (5'-3')** | **Reverse primer (5'-3')** |
| *Pa1* | TGGCCCAAACCTAAACATTAG | TTATGGCGCTTCATGTCTGAG |
| *Amh-cre* | CGAGTGATGAGGTTCGCAAG | CGTATATCCTGGCAGCGATC |

**Table S2**

| **RT-qPCR** |  |  |
| --- | --- | --- |
| **Gene** | **Forward primer (5'-3')** | **Reverse primer (5'-3')** |
| *Elmo1* | GAGAACAGCAGCCGAGAAGAT | GTTGCAGGTCTCACTAGGCAG |
| *Gdnf* | TCCAACTGGGGGTCTACGG | GCCACGACATCCCATAACTTCAT |
| *Tgfb3* | CCTGGCCCTGCTGAACTTG | TTGATGTGGCCGAAGTCCAAC |
| *Sfrp1* | CAACGTGGGCTACAAGAAGAT | GGCCAGTAGAAGCCGAAGAAC |
| *Hgf* | ATGTGGGGGACCAAACTTCTG | GGATGGCGACATGAAGCAG |
| *Met* | GTGAACATGAAGTATCAGCTCCC | TGTAGTTTGTGGCTCCGAGAT |
| *Cx43* | TTTCATTGGGGGAAAGGCGT | GCAGACTGTTCATCACCCCA |
| *Pa1* | GACTTTGATGATGAGCCAATGACAC | CTTATGGCGCTTCATGTCTGAGAG |
| *β-actin* | GGTGGGAATGGGTCAGAAGG | GTACATGGCTGGGGTGTTGA |
