# Supplementary material for: PA1 participates in the maintenance of blood–testis barrier integrity via cooperation with JUN in the Sertoli cells of mice
Source: Cell Biosci. 2022 Apr 4;12:41. doi: 10.1186/s13578-022-00773-y (PMC8981650; doi:10.1186/s13578-022-00773-y)
Supplement: Supplementary file 2 — Additional file 2. The detailed supplementary methods and materials mentioned in the article. [file 13578_2022_773_MOESM2_ESM.docx]

**Supplementary Material and Methods**

**Spermatocyte Surface Spreading**

Testes were decapsulated, and washed in PBS, pH 7.4, at room temperature. Then, tubules were immersed in a hypotonic buffer comprised of 30 mM Tris, 5 mM EDTA, 17 mM trisodium citrate dihydrate, 50 mM sucrose, 0.5 mM phenylmethylsulfonyl fluoride (PMSF) and 0.5 mM DTT (pH 8.2) for 0.5-1 h. Subsequently, the seminiferous tubules were teared in 100 mM sucrose, pH 8.2, on a glass slide by pipetting more than 20 times to make a cell suspension. The suspensions were mixed with spreading buffer containing 0.15% Triton X-100 and 1% paraformaldehyde (PFA), pH 9.2. The slides were dried overnight. After that, the dried slides were washed with PBS three times and blocked with 5% BSA. The slides were incubated with primary antibody at 4°C overnight, followed by incubation with the secondary antibody in the next day. The nuclei were further stained with DAPI. Images were taken using an LSM 780/710 microscope (Zeiss, Germany) or SP8 microscope (Leica, Germany).

**Immunofluorescence and Immunohistochemistry**

Testes from *Pa1^F/F^* and *Amh-pa1^-/-^* mice were fixed overnight in 4% PFA at 4°C, dehydrated through gradient ethanol, and embedded in paraffin. Mouse testes were taken and fixed in 4% PFA for 24-48 h, then dehydrated through gradient alcohol and xylene and embedded in paraffin. The 5 mm tissue sections were dewaxed, rehydrated, and antigen retrieval in 10 mM sodium citrate buffer (pH 6.0) for 15 min. After treatment with 0.1% Triton X-100 at room temperature for 10 mins, the sections were washed three times in PBS and blocked with 5% BSA. And the primary antibodies were added to the sections and incubated at 4°C overnight. In the next day, the sections were washed three times in PBS followed by incubation with the secondary antibody at 37°C for 1.5 h. Nuclei were stained with 40,6-diamidino-2-phenylindole (DAPI). For immunohistochemistry, after treatment with primary antibody, the sections were then incubated with 3% H_2_O_2_ to eliminate the activity of internal peroxidase. The sections were then incubated with a horseradish peroxidase (HRP)-labeled secondary antibody. Finally, the sections were reacted with 3,3’-diaminobenzidine (DAB), and further stained with hematoxylin. Images were captured using a Nikon inverted microscope with a CCD camera (Nikon, Eclipse Ti-S, Tokyo, Japan).

**RNA isolation, RT-qPCR, and Semi-RT-PCR**

Total cellular RNAs were isolated with the Trizol reagent (Invitrogen). cDNA was synthesized by the PrimeScriptTM RT Reagent Kit (TaKaRa, RR037A). Real-time qPCR was performed with a Roche Light Cycler® 480II System (WA, USA), and the results were analyzed using the LightCycle480 SW 1.5.1. Supplementary Table 2 contained the primer sequences used in RT-qPCR.

**Separation of Spermatogenic Cells**

In brief, testes of 8-week-old adult mice were decapsulated and digested in 10ml DMEM (Hyclone, Cat. No. SH30022.01B) containing 1.5 mg/ml Hyaluronidase, and 1.5 mg/ml collagenase IV (Sigma, C5138) at 37℃ for 15 min with gently shaking. The separated cells were then washed with DMEM, resuspended in 5ml DMEM and filtered via a 200-mesh filter (Solarbio, YA0961). The filtered cells were added to the top of the liquid in cell separation apparatus, followed by 600 ml of DMEM with a 2-4% BSA (Amresco, Solon, OH, AP0027) gradient. Different types of cells were collected from the bottom of the separator using the ordered tubules at a rate of 20 ml/min (10 ml/fraction) after 3 h of high-speed sedimentation under unit gravity. The purity and cell type in each tubule were assessed by light microscopy based on the size and morphological characteristics of the cells.

**Cut-Tag Experiments**

Sertoli cells were collected, counted, and centrifuged at 600 × g for 5 min at room temperature. Sertoli cells were washed twice with 1 mL Wash Buffer (-) (20 mM HEPES pH 7.5; 150 mM NaCl; 0.5 mM Spermidine; 1× Protease inhibitor cocktail) by gently pipetting. Con A coated magnetic beads (Vector, AL-1003-100) were prepared as described ^[29]^ and 5 μl of activated beads were used to bind with the cells at room temperature for 15 min. The beads-bound cells were incubated with primary antibody in 50 μl Wash (+) Buffer (20 mM HEPES pH 7.5; 150 mM NaCl; 0.5 mM Spermidine; Protease inhibitor cocktail; 0.05% Digitonin) together with 1mg/ml BSA on a rotating metal shaker at room temperature for 1 hour. With the magnet stand (12321D, Thermo Fisher Scientific), the supernatant was discarded and the cells were further incubated with secondary antibody (Guinea Pig anti-Rabbit IgG antibody, ABIN101961) in 50 μl of Wash buffer (+) and cells at room temperature for 1 h. After washed 3 times in 200 μl Wash buffer (+), the cells were further incubated with pA-Tn5 adapter product (151017, EpiCypher) in Tag Buffer (0.01% Digitonin, 20 mM HEPES, pH 7.5, 300 mM NaCl, 0.5mM Spermidine, 1× Protease inhibitor cocktail) at room temperature for 1 h. After the repeated washed for 3 times using 200 μl Wash buffer (+), cells were resuspended in 30 μl Activation buffer (10mM MgCl_2_ in Tag Buffer) and incubated at 37 °C for 1 h and further digested with 1.5 μl of 0.5 M EDTA, 0.3 μl of 10% SDS and 3 μl of 20 mg/mL Proteinase K at 55 °C for 1 hour followed by 65 °C for 1 hour. To extract the DNA, 40 μl SPRIselect® Reagent Kit (Beckman Coulter, Inc. #B23317) were used followed the protocol.

To construct the libraries, 23 μl purification products with 1 μl of a 10 μM i5 and 1μl of a 10 μM i7 primer were mixed and each sample is labeled with a unique group of i5 and i7 primer. Ultima Amplification Mix (Hieff NGS® Fast Tagment DNA Library Prep Kit for Illumina®, 12207-E, Yeasen) was added the sample was placed in a PCR machine following this cycling conditions: 72 °C for 3 min; 95 °C for 30 s; 14 cycles at 95 °C for 10 s, 55 °C for 30 s and 72 °C for 30 s; a final extension at 72 °C for 5 min; and holding at 4 °C. Post-PCR products were purified followed 1.2x SPRIselect® Reagent Kit (Beckman Coulter, Inc. #B23317). Paired-end Illumina sequencing of the barcoded libraries was performed using a NovaSeq 6000 System.
