# Supplementary figures and images for: PA1 participates in the maintenance of blood–testis barrier integrity via cooperation with JUN in the Sertoli cells of mice
Source: Cell Biosci. 2022 Apr 4;12:41. doi: 10.1186/s13578-022-00773-y (PMC8981650; doi:10.1186/s13578-022-00773-y)

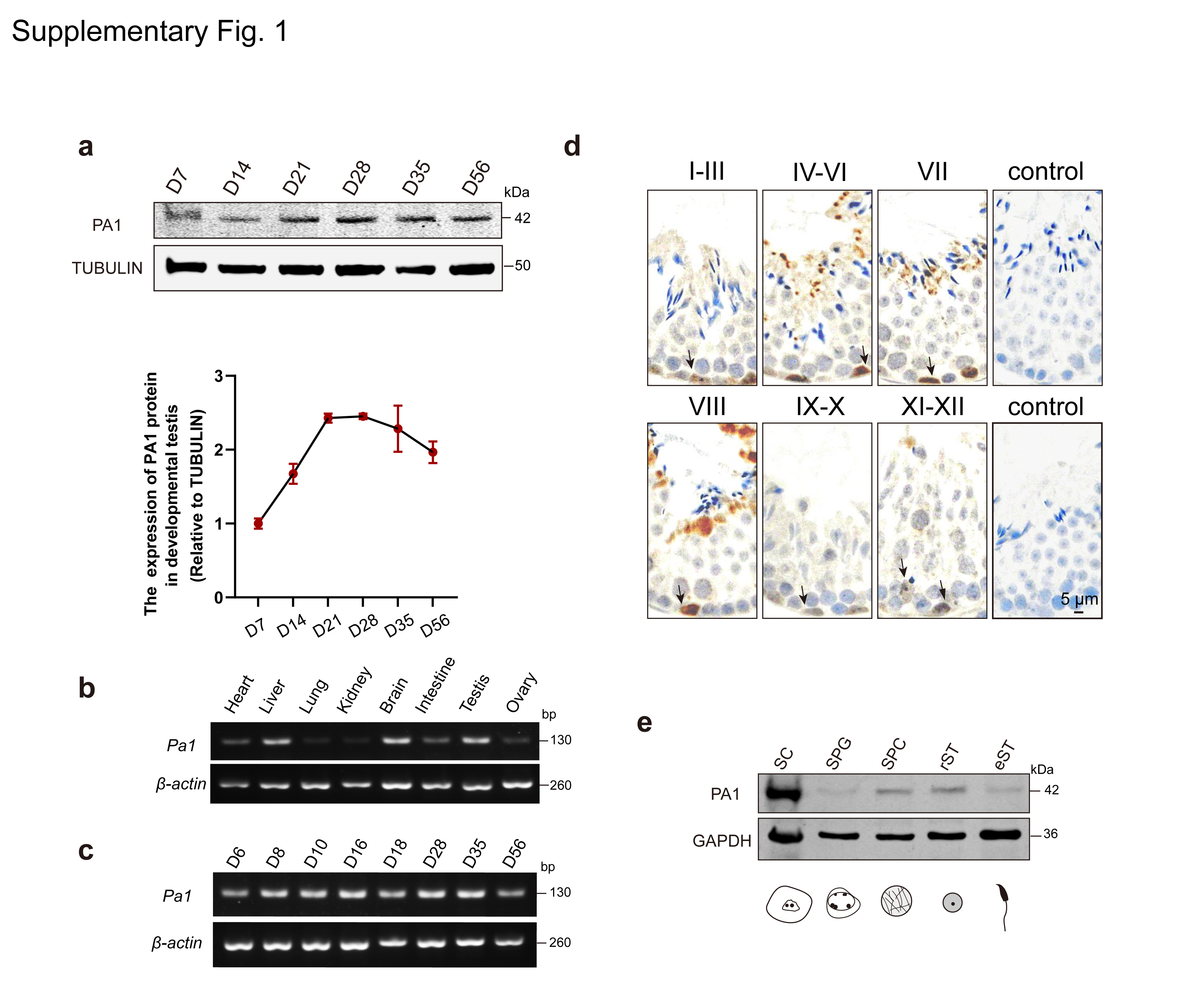

Supplement: Supplementary file 3 — Additional file 3: Fig. S1. The expression pattern of PA1 in human and mouse. a) PA1 protein levels gradually increased during the development of testis. The expression pattern of PA1 in different developmental stages of murine testes (Day 7, Day 14, Day 21, Day 28, Day 35, Day 56) using Western Blot. Statistical analysis of the expression level of PA1 during the development of testis was showed in the lower panel. b) Semi RT-PCR results showed Pa1 was ubiquitously expressed in different mouse organs but especially enriched in the testis. β-actin was used as the loading control. c) Semi RT-PCR results showed Pa1 levels gradually increased in mouse testis with the development of testis. β-actin was used as the loading control. d) PA1 is localized in Sertoli cells in mouse testis during the whole spermatogenic cycle while the expression level of PA1 in different stages varies. Testes sections from adult wild-type mice (8-week-old) were stained for PA1 using immunohistochemistry as indicated. Arrows indicate positive cells for PA1 expression. e) PA1 was expressed in different testicular cells but especially abundant in Sertoli cells. Immunoblotting of PA1 was performed in Sertoli cells, SC; spermatogonium, SPG; spermatocytes, SPC; round spermatids, rST; elongated and elongating spermatids, eST. [file 13578_2022_773_MOESM3_ESM.jpg]

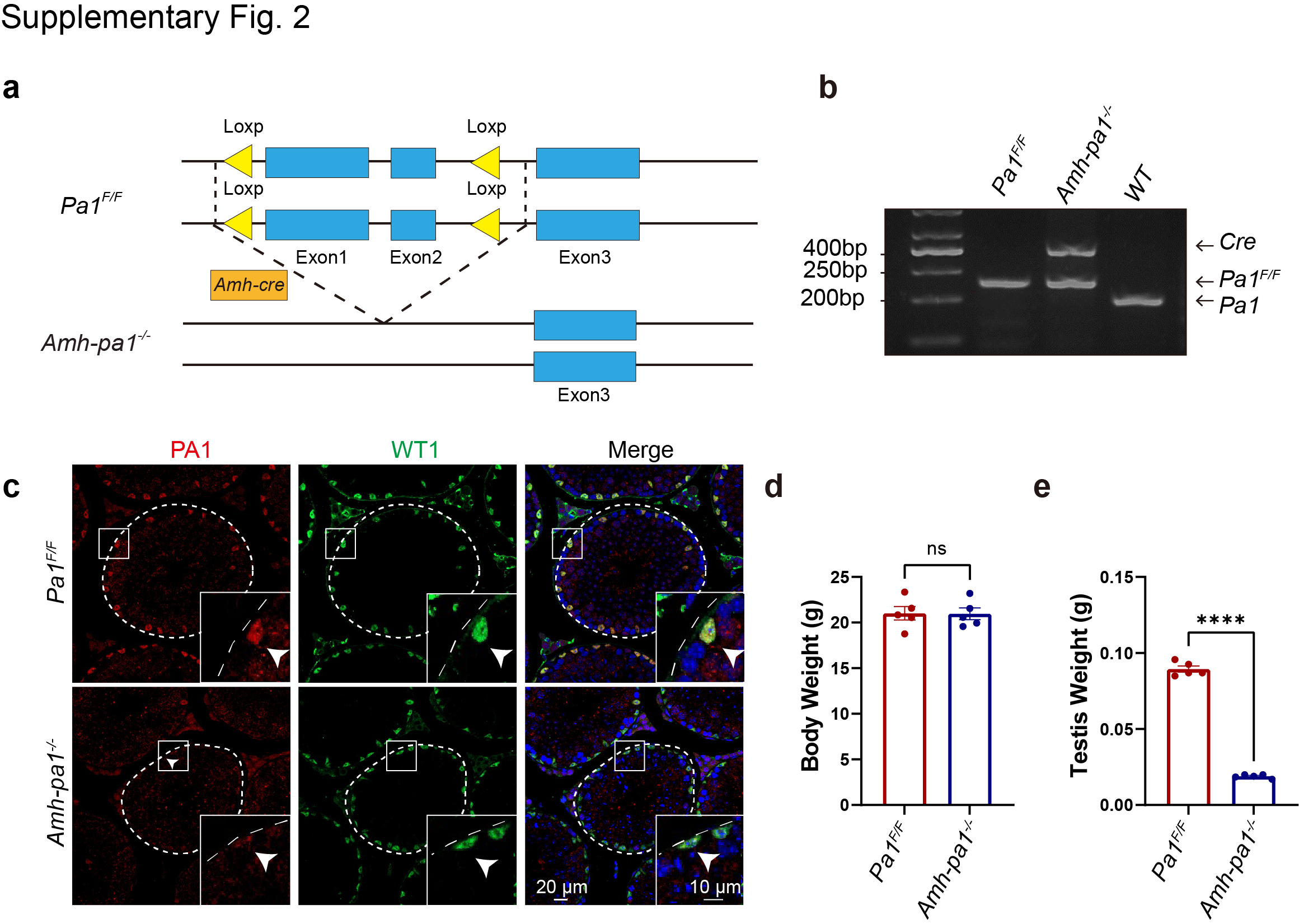

Supplement: Supplementary file 4 — Additional file 4: Fig. S2. Generation of Amh-pa1−/− mice. a) Schematic diagram for the generation of Amh-pa1−/− mice. Exon 1 and exon 2 (blue) of Pa1 were flanked by loxp sites (yellow) in Pa1F/F mice. These exons were specifically deleted in the Sertoli cells in Amh-pa1−/− mice. b) Amh-pa1−/−mice genotyping. The 250 bp band indicated floxed Pa1, the 200 bp band indicated Pa1, and the 400 bp band indicated Amh-cre. c) PA1 was specifically knocked out in testicular Sertoli cells of 5-week-old mice. Seminiferous tubule sections of Pa1F/F and Amh-pa1−/− testes were co-stained for WT1(green) and PA1 (red). The nuclei were stained with DAPI (blue). Solid boxes showed localization of the enlarged images. Dashed circle indicated basal membrane of seminiferous tubules. Arrows indicated the nuclei of Sertoli cells. d) Body weights of 8-week-old Pa1F/F mice and Amh-pa1−/− male mice (n = 5 independent experiments) were similar. Data are presented as mean ± SEM. e) Testis weights of Amh-pa1−/− mice were significantly lower than those of Pa1F/F mice (n = 5 independent experiments). Data are presented as mean ± SEM. ****p < 0.0001. [file 13578_2022_773_MOESM4_ESM.tif]

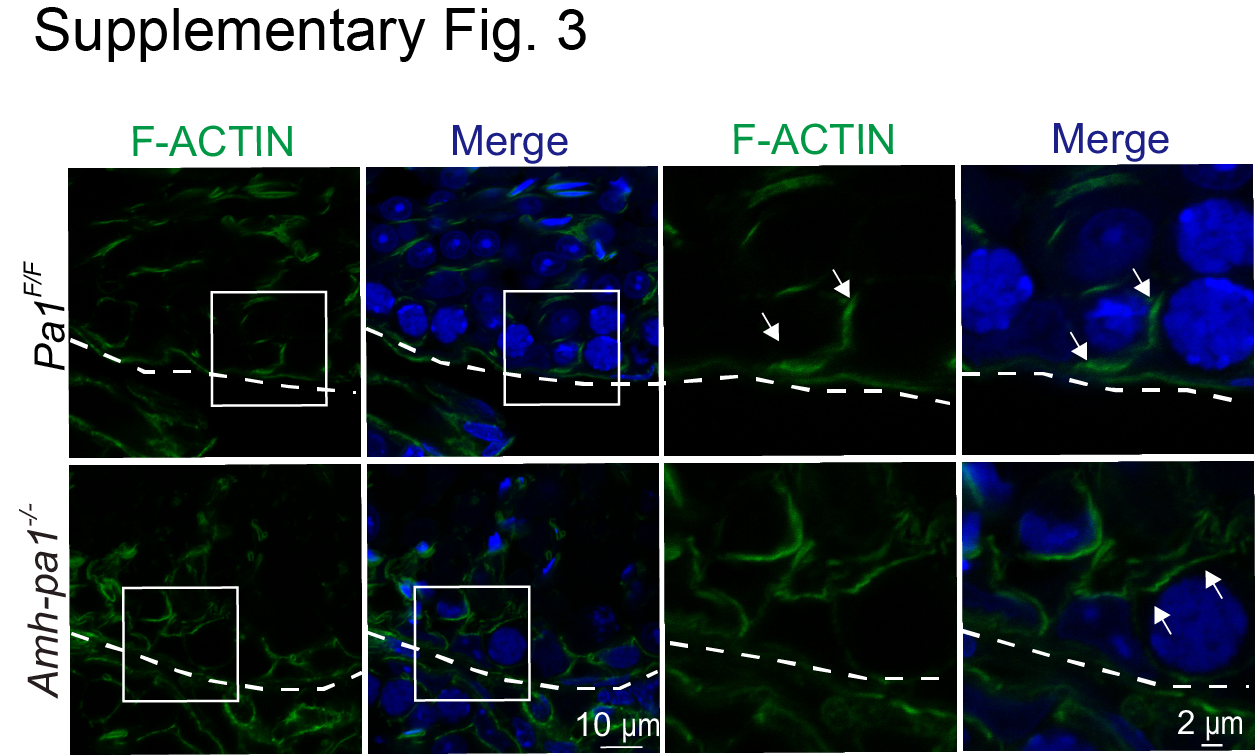

Supplement: Supplementary file 5 — Additional file 5: Fig. S3. Disorganized basal actin cytoskeleton is detected in Amh-pa1−/− mice testis. F-actin at the basal compartment of seminiferous tubules was observed to be perturbed and disorganized in adult Amh-pa1−/− testes. Arrows indicate F-actin bundles. Immunofluorescence using FITC-labeled phalloidin (green) was performed on the frozen testis sections of Pa1F/F mice (upper panels) and Amh-pa1−/− mice (lower panels). Nuclei were stained with DAPI (blue). Dashed boxes showed the localization of the enlarged images. Dashed curve indicated basal membrane of seminiferous tubules. [file 13578_2022_773_MOESM5_ESM.tif]

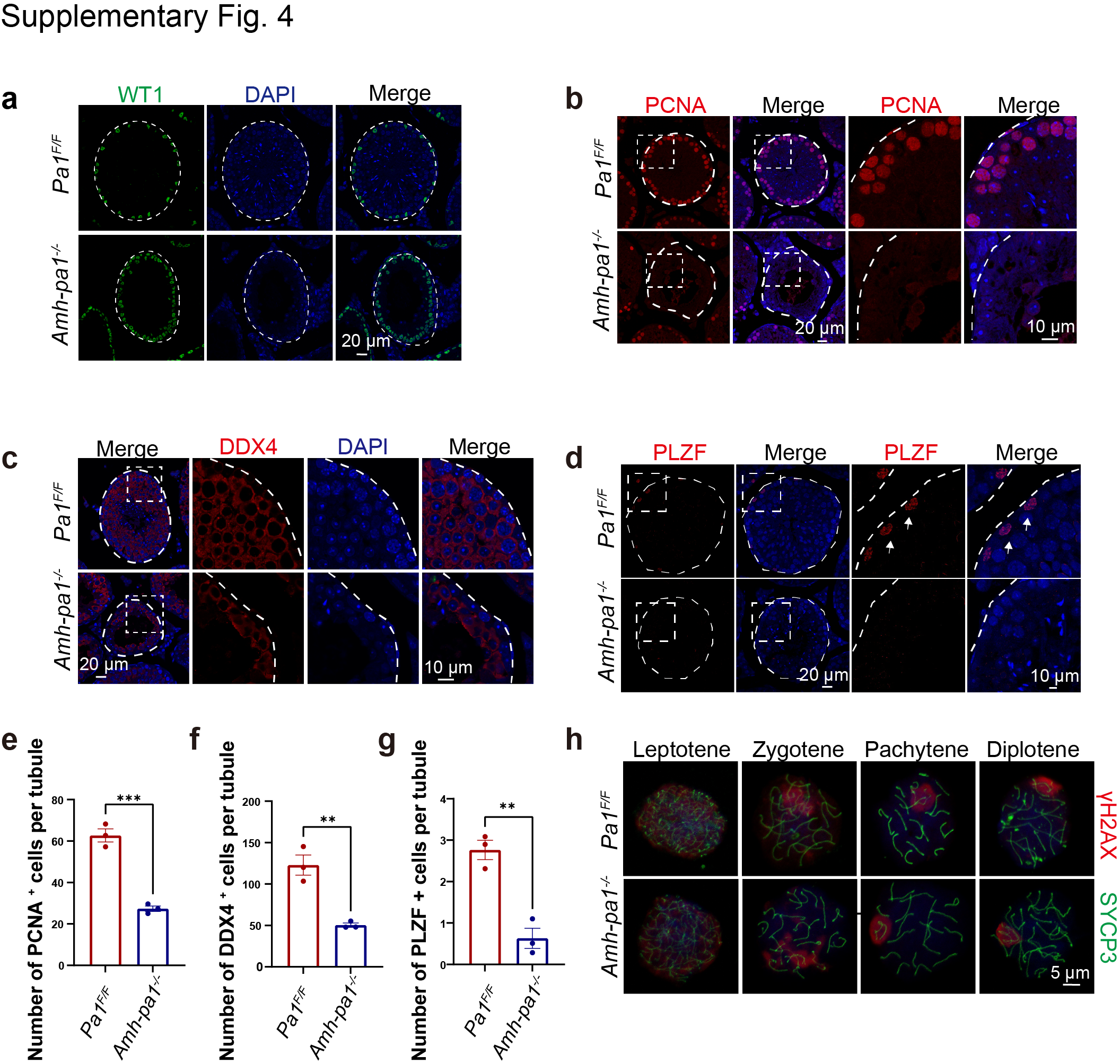

Supplement: Supplementary file 6 — Additional file 6: Fig. S4. Amh-pa1−/− mice show massive germ cell loss. a) Most of the residual cells in the empty tubules of Amh-pa1−/− testis were identified as Sertoli cells. Immunofluorescence analysis using anti-WT1 (green) was conducted on the sections of testis from Pa1F/F mice (upper panels) and Amh-pa1−/− mice (lower panels). Nuclei were stained with DAPI (blue). Dashed curve indicated basal membrane of seminiferous tubules. b) PCNA immunofluorescence analysis of seminiferous tubules showed that 8-week-old Amh-pa1−/− mice had decreased numbers of spermatocytes and spermatogonia compared with 8-week-old Pa1F/F mice. Seminiferous tubule sections of Pa1F/F and Amh-pa1−/− testes were stained for PCNA (red), and the nuclei was stained with DAPI (blue). Dashed boxes showed the localization of the enlarged images. Dashed curve indicated basal membrane of seminiferous tubules. Data are presented as mean ± SEM. ***p < 0.001. c) DDX4 immunofluorescence analysis of germ cells showed that 8-week-old Amh-pa1-/- mice had decreased numbers of germ cells compared with 8-week-old Pa1F/F mice. Seminiferous tubule sections of Pa1F/F and Amh-pa1−/− testes were stained for DDX4 (red), and the nuclei was stained with DAPI (blue). Dashed boxes showed the localization of the enlarged images. Dashed curve indicated basal membrane of seminiferous tubules. Data are presented as mean ± SEM. **p < 0.01. d) PLZF immunofluorescence analysis of germ cells showed that 8-week-old Amh-pa1−/− mice had decreased numbers of undifferentiated spermatogonium compared with 8-week-old Pa1F/F mice. Seminiferous tubule sections of Pa1F/F and Amh-pa1−/− testes were stained for PLZF (red), and the nuclei was stained with DAPI (blue). Dashed boxes showed the localization of the enlarged images. Dashed curve indicated basal membrane of seminiferous tubules. Arrows indicated PLZF positive cells. e) PCNA positive cell number per tubule was significantly decreased Amh-pa1−/− (27.34 ± 1.319) mice testes comp [file 13578_2022_773_MOESM6_ESM.tif]

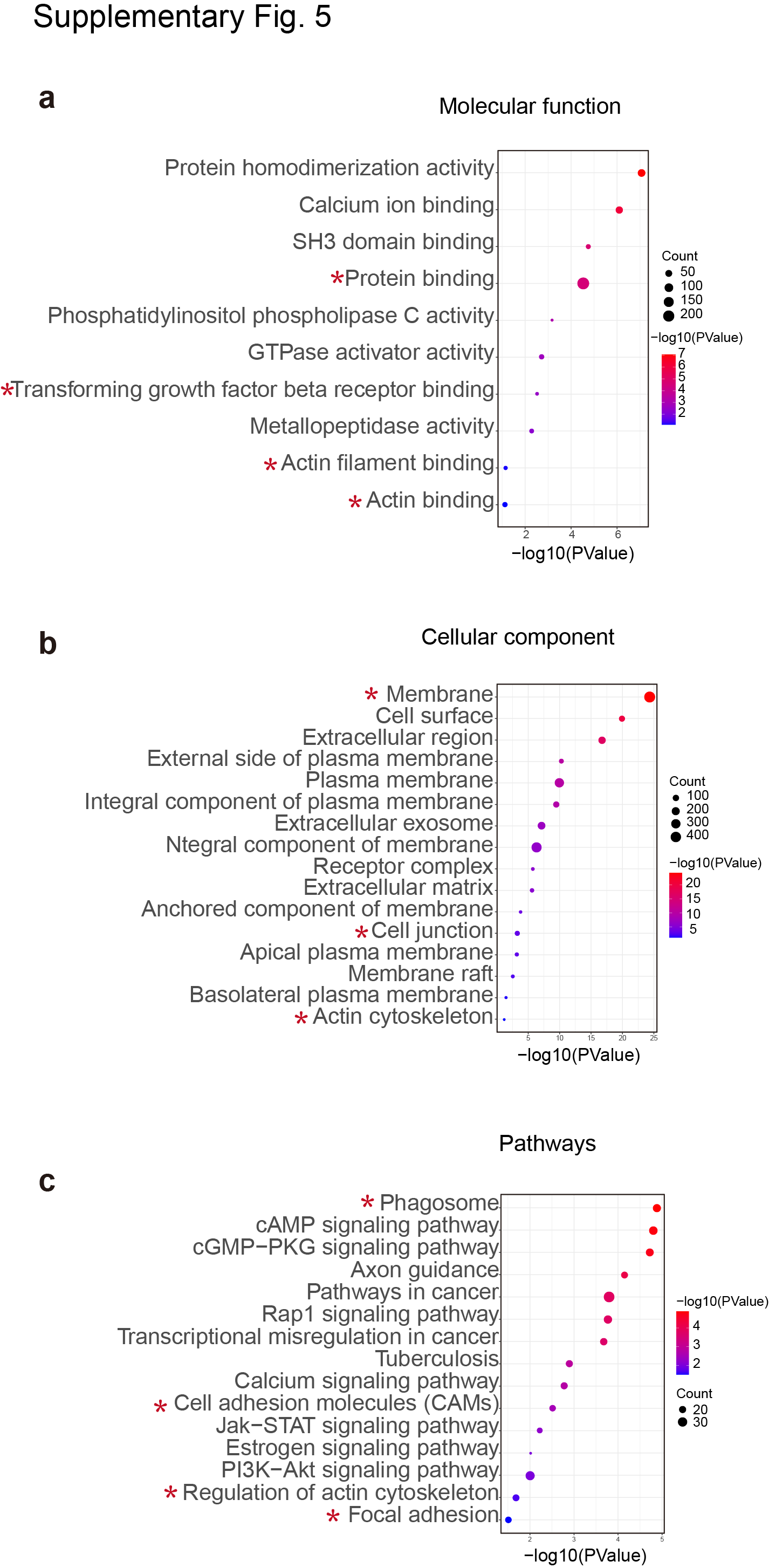

Supplement: Supplementary file 7 — Additional file 7: Fig. S5. 5 GO analysis of downregulated genes in Amh-pa1−/− Sertoli cells. a) GO-Molecular function analysis of 1045 downregulated genes in Amh-pa1−/− Sertoli cells. Molecular function of protein binding, actin filament binding and actin binding were labeled by asterisks. b) GO-Cellular component analysis of 1045 downregulated genes in Amh-pa1−/− Sertoli cells. Cellular component of membrane, cell junction and actin cytoskeleton were labeled with asterisks. c) KEGG pathway analysis of 1045 downregulated genes in Amh-pa1−/− Sertoli cells. KEGG pathways of phagosome, cell adhesion molecules, regulation of actin cytoskeleton and focal adhesion were labeled with asterisks. [file 13578_2022_773_MOESM7_ESM.tif]

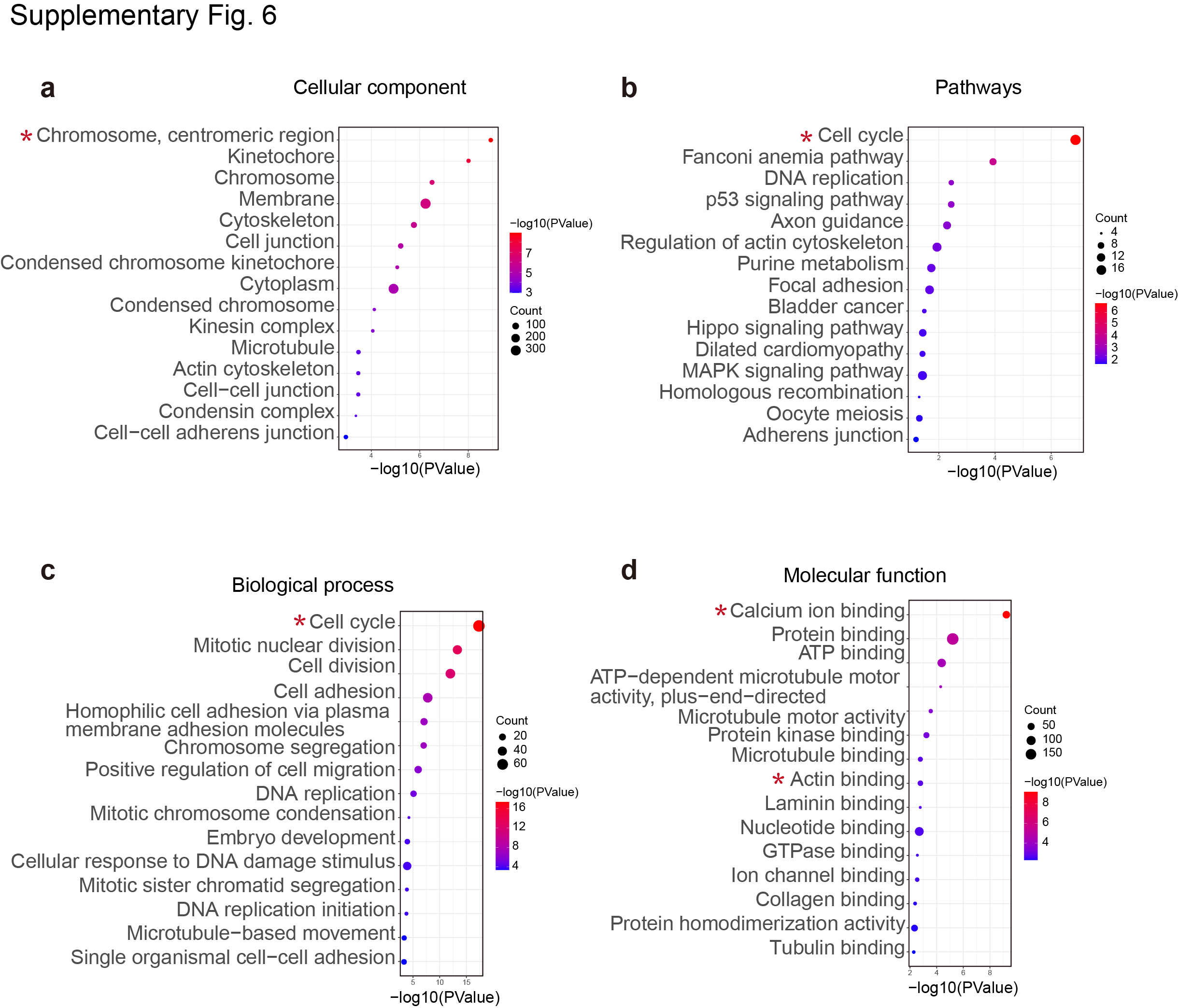

Supplement: Supplementary file 8 — Additional file 8: Fig. S6. GO analysis of upregulated genes in Amh-pa1−/− Sertoli cells. a) GO-Cellular component analysis of 899 upregulated genes in Amh-pa1−/− Sertoli cells. Cellular component of chromosome was labeled with asterisks. b) KEGG pathway analysis of 899 upregulated genes in Amh-pa1−/− Sertoli cells. KEGG pathways of cell cycle was labeled with asterisks. c) GO-Biological process analysis of 899 upregulated genes in Amh-pa1−/− Sertoli cells. Biological process of cell cycle was labeled with asterisks. d) GO-Molecular function analysis of 899 upregulated genes in Amh-pa1−/− Sertoli cells. Molecular function of calcium ion binding, and actin binding were labeled by asterisks. [file 13578_2022_773_MOESM8_ESM.tif]

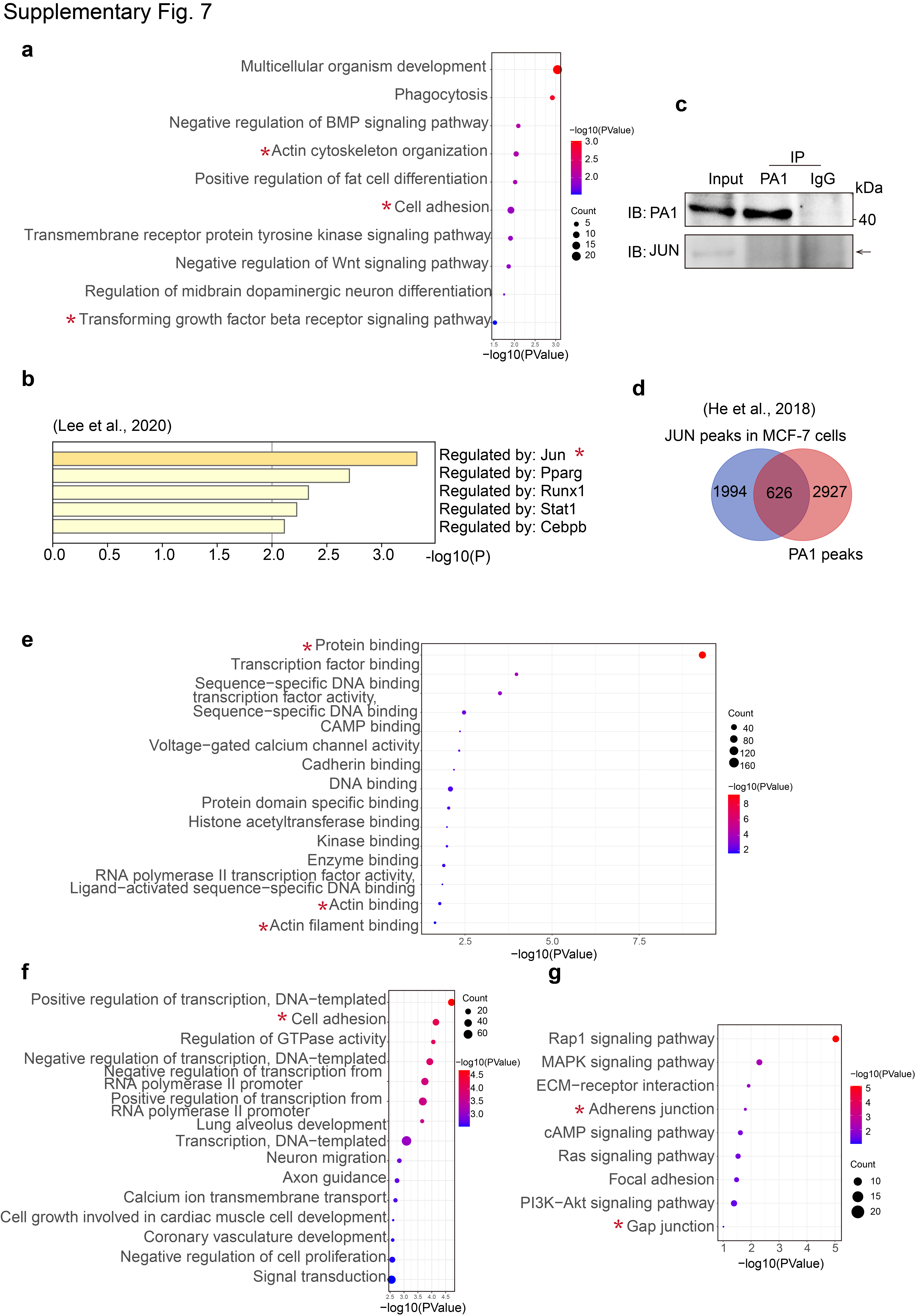

Supplement: Supplementary file 9 — Additional file 9: Fig. S7. PA1 together with JUN regulates downstream gene expression. a) GO-Biological process analysis of the 184 overlapped genes identified in both downregulated genes of Amh-pa1−/− Sertoli cells and PA1 bound genes. Biological process of actin cytoskeleton organization, cell adhesion and transforming growth factor beta receptor signaling pathway were labeled with asterisks. b) Upstream transcription factor analysis of downregulated genes in Pa1-knockout MEFs. The identified top 1 transcription factor JUN was labeled with asterisk. c) The endogenous co-immunoprecipitation results of PA1 and JUN in TM4 cells. The proteins of TM4 cells were collected and immunoprecipitated using anti-PA1 antibody and the products were further used for immunoblot analysis with anti-PA1 and anti-JUN antibodies. The bands for PA1 and JUN were labeled with the arrows. d) Venn diagram showing the overlap of PA1 bound genes in Sertoli cells with JUN bound genes in MCF-7 cells [43]. e) GO-Molecular function analysis of the 626 overlapped genes identified in both PA1 bound genes in Sertoli cells and JUN bound genes in MCF-7 cells [43]. Molecular function of protein binding, actin binding and actin filament binding were labeled with asterisks. f) GO-Biological process analysis of the 626 overlapped genes identified in both PA1 bound genes in Sertoli cells and JUN bound genes in MCF-7 cells [43]. Biological process of cell adhesion was labeled with asterisks. g) KEGG pathway analysis of the 626 overlapped genes identified in both PA1 bound genes in Sertoli cells and JUN bound genes in MCF-7 cells. KEGG pathway of adherens junction and gap junction were labeled with asterisks. [file 13578_2022_773_MOESM9_ESM.tif]
